# Effect of long‐term caloric restriction on telomere length in healthy adults: CALERIE™ 2 trial analysis

**DOI:** 10.1111/acel.14149

**Published:** 2024-03-19

**Authors:** Waylon J. Hastings, Qiaofeng Ye, Sarah E. Wolf, Calen P. Ryan, Sai Krupa Das, Kim M. Huffman, Michael S. Kobor, William E. Kraus, Julia L. MacIsaac, Corby K. Martin, Susan B. Racette, Leanne M. Redman, Daniel W. Belsky, Idan Shalev

**Affiliations:** ^1^ Department of Psychiatry and Behavioral Sciences Tulane University School of Medicine New Orleans Louisiana USA; ^2^ Department of Biobehavioral Health Pennsylvania State University, University Park State College Pennsylvania USA; ^3^ Institute for Ecology and Evolution, School of Biological Sciences University of Edinburgh Edinburgh UK; ^4^ Butler Columbia Aging Center Columbia University Mailman School of Public Health New York New York USA; ^5^ Jean Mayer USDA Human Nutrition Research Center on Aging at Tufts University Boston Massachusetts USA; ^6^ Duke Molecular Physiology Institute and Department of Medicine Duke University School of Medicine Durham North Carolina USA; ^7^ Edwin S.H. Leong Centre for Healthy Aging, Department of Medical Genetics University of British Columbia Vancouver British Columbia Canada; ^8^ Pennington Biomedical Research Center Baton Rouge Louisiana USA; ^9^ College of Health Solutions Arizona State University Phoenix Arizona USA; ^10^ Department of Epidemiology Columbia University Mailman School of Public Health New York New York USA

**Keywords:** caloric restriction, geroscience, randomized clinical trial, telomere length

## Abstract

Caloric restriction (CR) modifies lifespan and aging biology in animal models. The Comprehensive Assessment of Long‐Term Effects of Reducing Intake of Energy (CALERIE™) 2 trial tested translation of these findings to humans. CALERIE™ randomized healthy, nonobese men and premenopausal women (age 21–50y; BMI 22.0–27.9 kg/m^2^), to 25% CR or ad‐libitum (AL) control (2:1) for 2 years. Prior analyses of CALERIE™ participants' blood chemistries, immunology, and epigenetic data suggest the 2‐year CR intervention slowed biological aging. Here, we extend these analyses to test effects of CR on telomere length (TL) attrition. TL was quantified in blood samples collected at baseline, 12‐, and 24‐months by quantitative PCR (absolute TL; aTL) and a published DNA‐methylation algorithm (DNAmTL). Intent‐to‐treat analysis found no significant differences in TL attrition across the first year, although there were trends toward increased attrition in the CR group for both aTL and DNAmTL measurements. When accounting for adherence heterogeneity with an Effect‐of‐Treatment‐on‐the‐Treated analysis, greater CR dose was associated with increased DNAmTL attrition during the baseline to 12‐month weight‐loss period. By contrast, both CR group status and increased CR were associated with reduced aTL attrition over the month 12 to month 24 weight maintenance period. No differences were observed when considering TL change across the study duration from baseline to 24‐months, leaving it unclear whether CR‐related effects reflect long‐term detriments to telomere fidelity, a hormesis‐like adaptation to decreased energy availability, or measurement error and insufficient statistical power. Unraveling these trends will be a focus of future CALERIE™ analyses and trials.

## Graphical abstract

We found mixed evidence for effects of 2‐year moderate caloric restriction (CR) on telomere length (TL). Although CR was associated with accelerated TL attrition in Year 1, sustained CR without weight loss was associated with reduced TL attrition across Year 2. As a result, there were no differences in TL attrition from baseline to intervention conclusion at 24 months, leaving it unclear whether short‐term dynamics reflect hormetic adaptation to decreased energetic intake or measurement error and low statistical power.

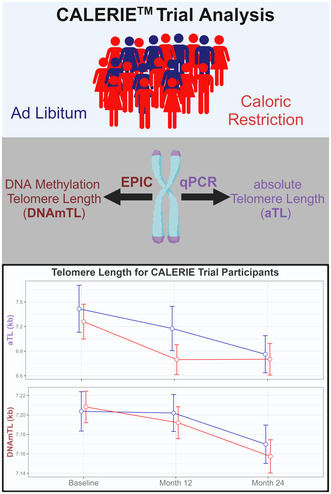

**Abbreviations:**
AL: Ad Libitum
aTL: Absolute Telomere Length
BMI: Body Mass Index
CALERIE™: Comprehensive Assessment of Long‐Term Effects of Reducing Intake of Energy
CI: Confidence Interval
CpG: 5′ – Cytosine‐Phosphate‐Guanine – 3′
CR: Caloric Restriction
DNA: Deoxyribonucleic Acid
DNAmTL: DNA Methylation Estimated Telomere Length
DunedinPACE: Dunedin Pace of Aging Calculated from the Epigenome
EPIC: Infinium MethylationEPIC BeadChip
ICC: Intraclass Correlation Coefficient
ITT: Intent to Treat
IV: Instrumental Variable
NIA: National Institute on Aging
PC: Principal Component
PREDIMED: Prevencion con Dieta Mediterranea
qPCR: Quantitative Polymerase Chain Reaction
RCT: Randomized Control Trial
SHINE: Stroke Hyperglycemia Insulin Network Effort
TEE: Total Energy Expenditure
TL: Telomere Length
TOT: Effect of Treatment on the Treated

## 1 INTRODUCTION

Caloric restriction (CR) extends average and maximum lifespans in a variety of species, from yeast to primates (Anderson & Weindruch, 2012; Colman et al., 2014). The foremost data for the effects of CR in humans comes from the NIA‐supported Comprehensive Assessment of the Long‐Term Effects of Reducing Energy (CALERIE™) 2 study (Rochon et al., 2011). Prior results from CALERIE™ 2 provide evidence for significant improvements to quality of life, cardiometabolic integrity, liver functioning, skeletal muscle quality, and immune health following moderate CR (11.9 ± 0.7%) over a span of 2 years (Das et al., 2023; Dorling et al., 2021; Kraus et al., 2019; Martin et al., 2016; Meydani et al., 2016). CR in CALERIE™ also reduced the rate of biological aging measured with a panel of clinical biomarkers representing physiological integrity across multiple organ systems (e.g., creatinine, albumin, blood glucose, blood pressure) (Belsky et al., 2017).

Evidence is mixed for genomic phenotypes of aging. Gene expression analyses in subsets of CALERIE™ participants suggest CR modulates core longevity pathways related to mitochondrial stability, inflammation, and oxidative stress (Das et al., 2023; Spadaro et al., 2022). In contrast, epigenetic clock analyses yielded mixed results, with the DunedinPACE clock, which was designed to measure the pace of aging (Belsky et al., 2022), indicating slowed aging in response to CR while other epigenetic clocks showed no response (Waziry et al., 2023).

Processes of mitochondrial stability, inflammation, and oxidative stress are also implicated in telomere length (TL) regulation (Shalev & Hastings, 2019). Most somatic cells undergo programmed telomere shortening, eventually leading to DNA damage response and cell cycle arrest. In this manner, TL attrition is purported to be a hallmark of biological aging (Lopez‐Otin et al., 2013). Importantly, correlations between TL, blood chemistry indices, and epigenetic clock measures of biological aging are generally low (Belsky et al., 2018; Hastings et al., 2019), suggesting different measurement approaches may capture distinct aspects of the aging process. In laboratory experiments, lifetime CR reduces the rate of telomere attrition in rats (Pendergrass et al., 2001) and mice (Vera et al., 2013). In humans, cross‐sectional data showing an inverse association between energy intake and TL provide indirect support of similar CR intervention effects (Kark et al., 2012), as does evidence of TL lengthening observed in obese individuals maintaining 5% or greater weight loss over a 1 year period (Mason et al., 2018). Nonetheless, whether CR slows biological aging as indicated by the rate of TL attrition has not been tested in healthy, nonobese humans.

**FIGURE 1:**
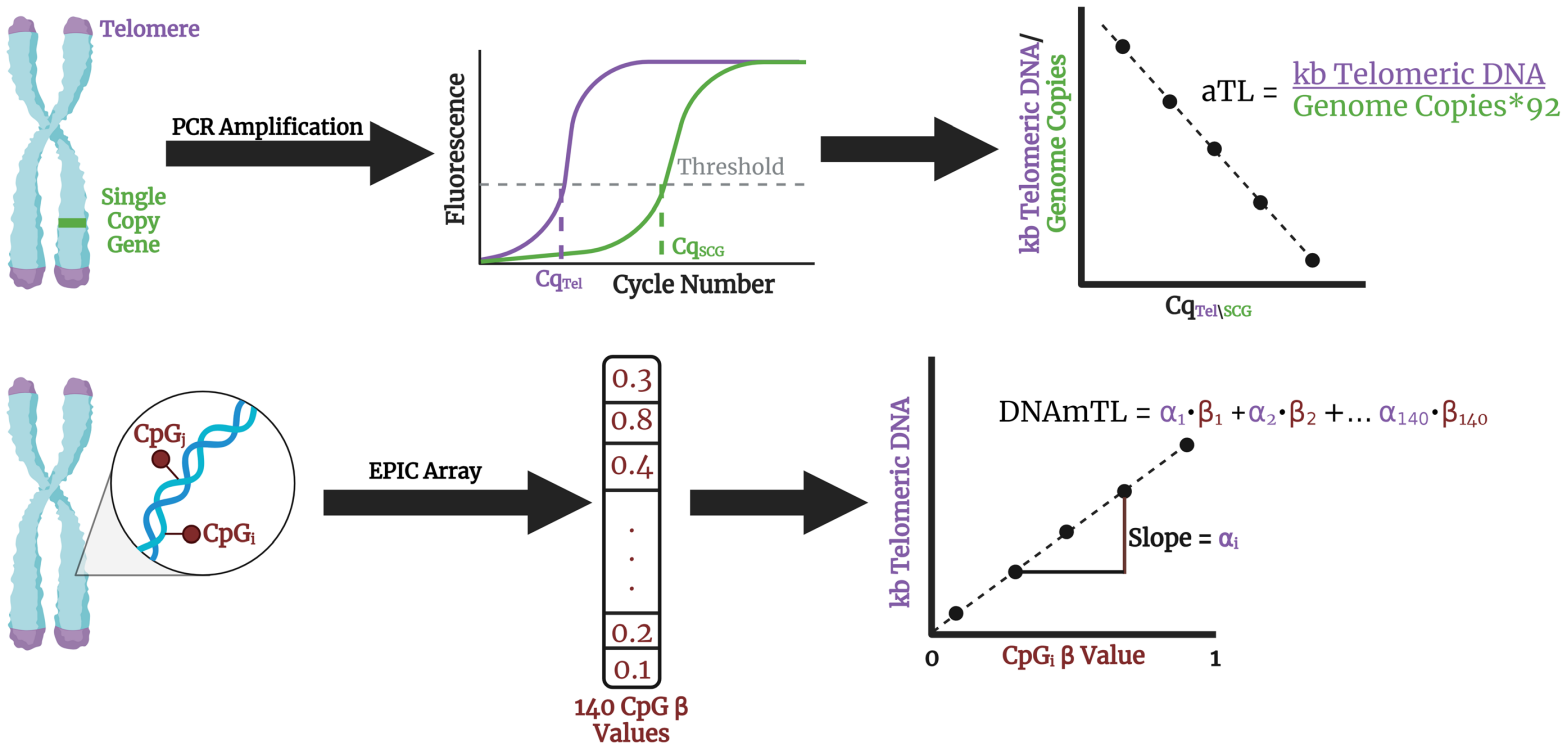
Telomere Length Assessment in CALERIE™. Assessment of aTL with qPCR involves quantifying levels of telomeric DNA content relative to the number of genomes using the single copy gene *IFNB1* encoding interferon 1 beta. In this approach, the number of cycles a sample takes to reach a threshold of fluorescence detection, the Cq or cycle of quantification, is inversely proportional to the amount of target DNA present at baseline. Using oligomers with known amounts of telomeric DNA and single copy gene sequence, we constructed a standard curve to estimate the kb per telomere. Alternatively, TL can be estimated from DNA methylation assessed using the EPIC v1 array. In this approach, we extracted 140 probe (CpG) beta values, each representing the proportion of reads where that CpG site was methylated, and estimated DNAmTL using previously documented associations between each probe and TL measured using the Southern blot analysis of terminal restriction fragment lengths.

Building on previous analyses with systemic biomarker indices (Belsky et al., 2017), gene expression (Das et al., 2023; Spadaro et al., 2022), and epigenetic clocks (Waziry et al., 2023), the aim of the current study was to test the hypothesis that CR modulates biological aging in humans via slowing the rate of TL attrition. We used two approaches for TL measurement, direct assessment of absolute telomere length (aTL) using quantitative PCR (qPCR) (O'Callaghan & Fenech, 2011), and indirect estimation with the DNA methylation‐based estimator of telomere length (DNAmTL) (Lu et al., 2019), both of which provide measurements in kilobase pair (kb) units (Figure 1). Although these measures trace the same biological phenomena, they are only moderately correlated, suggesting they may capture distinct effects of CR on TL attrition (Doherty et al., 2023; Hastings et al., 2022; Lu et al., 2019).

## 2 RESULTS

### 2.1 Sample demographics

The analysis sample included a total of 175 participants who had data for both TL measurement methods at baseline and at least one follow‐up assessment (12 and/or 24 months). Of these, 54 ad libitum (AL) participants (86%) and 93 CR participants (83%) had aTL as well as DNAmTL data for all three time points. A summary of participant demographics at baseline is presented in Table S1. There were no significant differences between groups for any baseline characteristics. There were also no significant differences between analysis sample and remaining CALERIE™ participants (Table S2).

### 2.2 TL characterization

**FIGURE 2:**
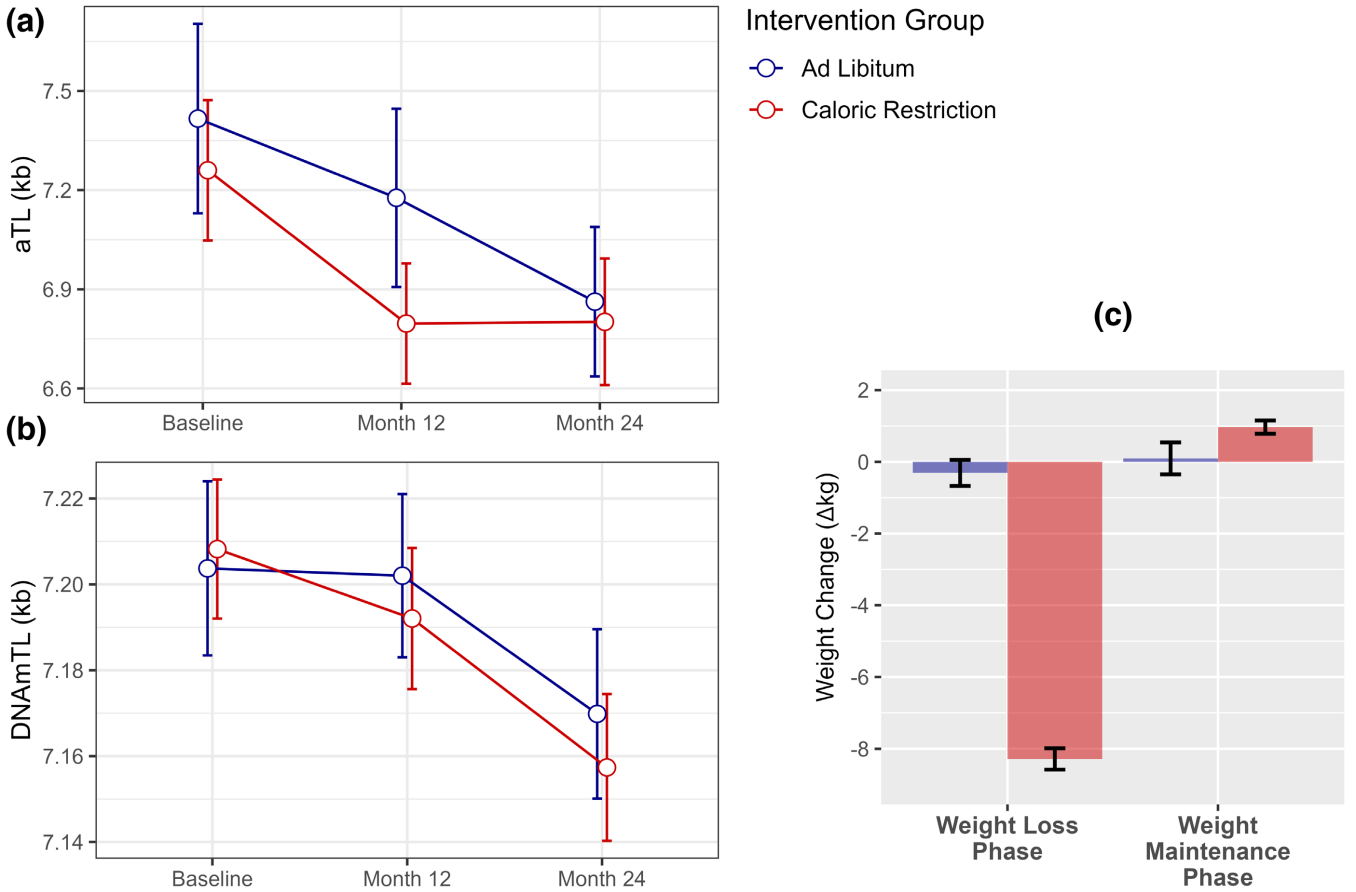
CALERIE™ participant TL and weight change throughout the intervention period. (a). Telomere length measured using qPCR (aTL). (b). Telomere length estimated using DNA methylation (DNAmTL). Data points are offset slightly for ease of visualization. (c). Participant weight change between baseline and 12‐month assessments (Weight Loss Phase), and between 12‐ and 24‐month assessments (Weight Maintenance Phase). Experimental groups distinguished by color (blue = AL; red = CR). Error bars represent standard error of the mean.

TL measures exhibited moderate agreement. aTL and DNAmTL measurements were significantly correlated with each other across all time points (*r* = 0.26–0.29, Table S3), in accordance with prior work (Doherty et al., 2023; Hastings et al., 2022). On average, aTL measurements were 166 bp shorter than DNAmTL estimates (*t*_546_ = −1.91, *p* = 0.057). aTL measurements were longer at baseline (μ_Diff_ = 109 bp, *t*_174_ = 0.66, *p* = 0.512), but were shorter at 12‐months (μ_Diff_ = −265 bp, *t*_161_ = −1.78, *p* = 0.076) and significantly shorter at 24‐months (μ_Diff_ = −338 bp, *t*_159_ = −2.355, *p* = 0.020). Mean values of TL measures at baseline and follow‐up assessments are plotted in Figure 2 and reported in Table S4. A Bland–Altman plot of DNAmTL and aTL measurements is provided in Figure S1.

TL measures replicated known associations with age and race (Table 1, see Table S5 for models with standardized effect sizes). Both measures significantly decreased with chronological age (Δ_aTL_ = 87 bp/year; Δ_DNAmTL_ = 17 bp/year) and were longer in participants identifying as Black as compared to White (Δ_aTL_ = 1.18 kb; Δ_DNAmTL_ = 62 bp). Neither aTL nor DNAmTL was associated with BMI stratum at randomization. Associations with other demographic factors varied by measurement approach. Significant sex differences were observed for DNAmTL measures only, but in the direction opposite expectation, with females exhibiting 42 bp shorter average DNAmTL than males. aTL measurements were longer in females but did not reach statistical significance. Effects were consistent across baseline 12, and 24 months, except for racial differences in aTL, which were not significant at baseline assessments (Table S6). Results remained unchanged following additional covariate control for immune cell proportions (Tables S7 and S8).

**TABLE 1:** General linear models testing associations between sample demographics and TL measurements.

	aTL			DNAmTL		
	*β*	[95% CI]	*p*	*β*	[95% CI]	*p*
Intercept	10.18	[8.56, 11.80]	**<0.001**	7.90	[7.80, 7.99]	**<0.001**
Age	−0.09	[−0.12, −0.05]	**<0.001**	−0.02	[−0.02, −0.02]	**<0.001**
Sex	0.29	[−0.32, 0.91]	0.347	−0.04	[−0.08, −0.01]	**0.027**
BMI: Overweight	−0.11	[−0.67, 0.45]	0.704	0.01	[−0.02, 0.04]	0.526
Race: Black	1.18	[0.33, 2.03]	**0.007**	0.06	[0.01, 0.11]	**0.019**
Race: Other	0.49	[−0.36, 1.35]	0.257	−0.02	[−0.07, 0.04]	0.556

*Note*: Coefficient for age reflects kb difference in TL for each one‐year increase in chronological age. Coefficients for BMI, Sex, and Race are kb difference relative to reference groups of Lean BMI, males, and Whites. Significant effects indicated in bold. Results represent associations between TL measurements aggregated from all study time points. Results for individual assessments (i.e., baseline, 12‐months, & 24‐months) are presented in Table S5.

We also investigated associations between TL and processes of oxidative stress and inflammation. Oxidative stress and inflammation levels decreased over time and tended to be lower in the CR group (Table S9), in line with previous reports (Il'yasova et al., 2018; Meydani et al., 2016). Contrary to expectations, inflammation factor scores were positively associated with longer aTL across the study time points (Table S10). Discriminate analyses using individual markers of inflammation (Table S11) revealed similar associations between leptin and aTL (*p* = 0.045). Although oxidative stress levels were not associated with either TL measurement (Table S10), analysis of individual F2‐isoprostanes revealed significant associations between aTL and iPF2α‐III, a common measure of lipid peroxidation (Basu, 2008). These associations were in the hypothesized direction, such that increased levels of iPF2α‐III were associated with shorter aTL (Table S12).

### 2.3 Impact of CALERIE™ parameters on TL

We conducted analyses using TL change scores quantifying the within‐person kb difference between baseline and 12 and/or 24 months. Mean values of TL change scores are reported in Table S13.

TL change scores between baseline and 12‐ and 24‐ month assessments were not significantly different between CR and AL intervention groups (Table S13), although there were trends toward increased attrition in the CR group between baseline and 12 months for both measurement approaches (*p*_aTL_ = 0.056; *p*_DNAmTL_ = 0.068). By contrast, CR group aTL change scores were significantly smaller during the Weight Maintenance Phase between 12‐ and 24‐month assessments (*p* = 0.027), reflecting null changes in average aTL for the CR group across this duration (Figure 2, Table S4). Similar findings were observed in intent to treat (ITT) analyses (Table 2, Panel A).

**TABLE 2:** Models testing the impact of caloric restriction on change in TL.

	aTL			DNAmTL		
	*β*	[95% CI]	*p*	*β*	[95% CI]	*p*
Panel A: Intent to treat						
Baseline to 12 Months	0.371	[−0.03, 0.77]	0.068	0.017	[0.00, 0.03]	0.054
Baseline to 24 Months	−0.151	[−0.55, 0.24]	0.453	0.011	[−0.01, 0.03]	0.192
Maintenance Phase	−0.490	[−0.90, −0.08]	**0.020**	−0.004	[−0.02, 0.01]	0.652
Panel B: Dose–response						
Baseline to 12 Months (*low CR*)	0.363	[−0.20, 0.93]	0.205	0.016	[−0.01, 0.04]	0.199
Baseline to 24 Months (*low CR*)	−0.099	[−0.60, 0.40]	0.697	0.004	[−0.02, 0.02]	0.717
Maintenance phase (*low CR*)	−0.454	[−0.97, 0.06]	0.084	−0.013	[−0.04, 0.01]	0.229
Baseline to 12 Months (*high CR*)	0.374	[−0.05, 0.80]	0.084	0.017	[0.00, 0.03]	0.066
Baseline to 24 Months (*high CR*)	−0.186	[−0.63, 0.26]	0.411	0.016	[0.00, 0.03]	0.096
Maintenance Phase (*high CR*)	−0.515	[−0.98, −0.05]	**0.030**	0.002	[−0.02, 0.02]	0.817
Panel C: Effect of treatment on the treated (TOT)						
Baseline to 12 Months	0.535	[−0.010, 1.079]	0.056	0.026	[0.003, 0.050]	**0.031**
Baseline to 24 Months	−0.126	[−0.810, 0.559]	0.655	0.022	[−0.006, 0.049]	0.125
Maintenance Phase	−0.856	[−1.684, −0.027]	**0.045**	−0.014	[−0.050, 0.021]	0.433

*Note*: Coefficients in Panel A and Panel B reflect kb difference in TL attrition between baseline and each follow up assessment associated with CR intervention group status. Maintenance Phase coefficients refer to associations with TL attrition between 12‐ and 24‐ month follow up assessments. Coefficients in Panel C‐F reflect kb difference in TL attrition associated with a 20% increase in CR, independent of group status. Positive values reflect faster TL attrition between assessments, while negative values reflect slower TL attrition between assessments. Significant effects indicated in bold.

Since average %CR in the CALERIE™ trial was below the prescribed 25% (mean = 11.9, SE = 0.7%), we conducted dose–response analyses to test if higher CR doses elicited larger treatment effects. Following the approach in previous work (Belsky et al., 2017; Waziry et al., 2023), we stratified the CR group by whether participants had higher (> = 10%) or lower (<10%) CR, and repeated ITT analyses testing the intervention effects in each stratified CR group against the AL controls (Table 2, Panel B). The CR intervention was not associated with change in TL for those with low adherence. Intervention effects across 12‐ and 24‐months were also not significant for those with high adherence, although there were once again trends toward increased TL attrition between baseline and 12 months relative to AL participants (*p* = 0.084 for aTL and *p* = 0.066 for DNAmTL). Highly adherent participants were also characterized by significantly less aTL attrition in the Maintenance Phase between 12 and 24 months (*p* = 0.030).

**FIGURE 3:**
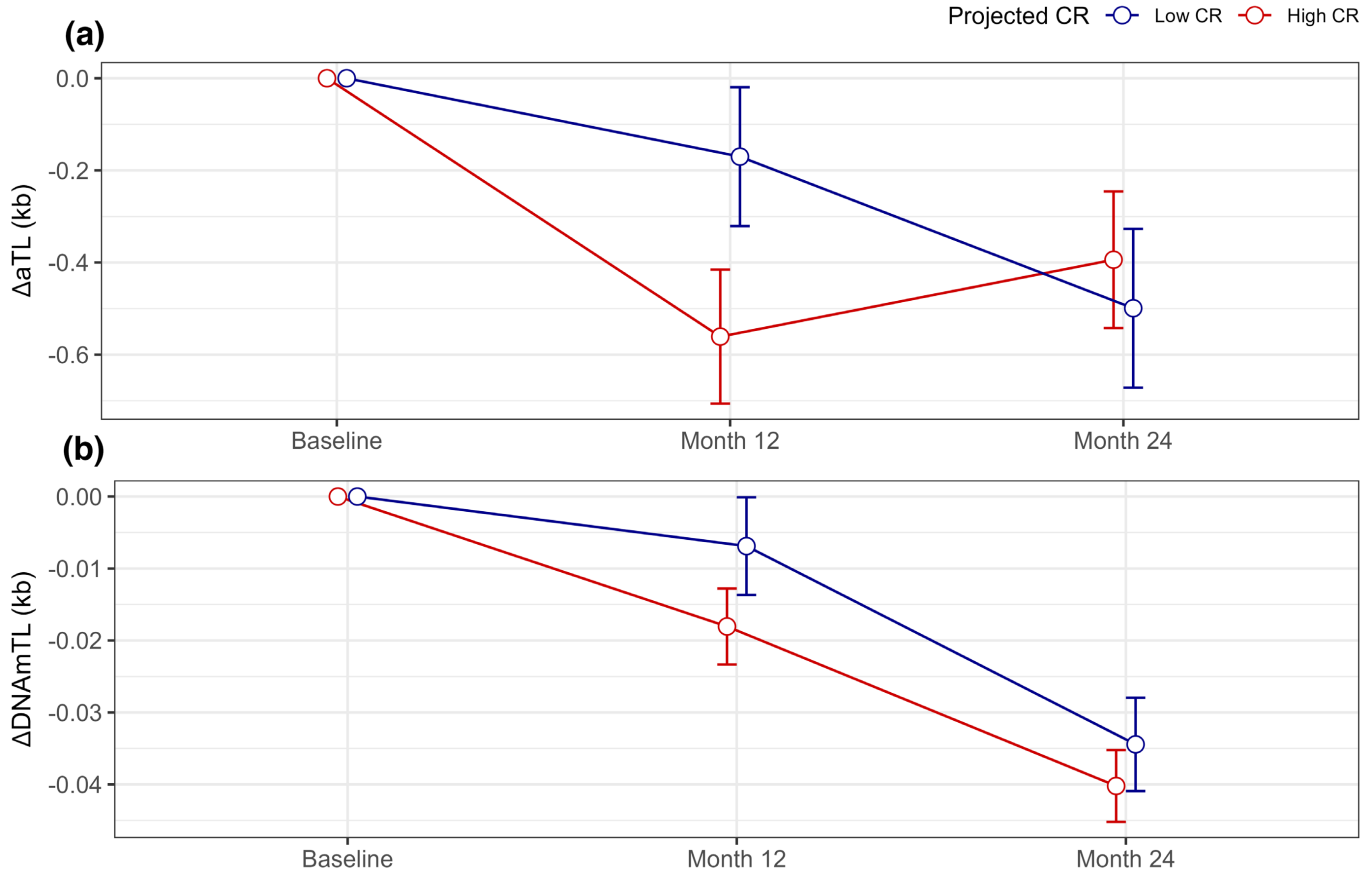
CALERIE™ participant change in TL derived during TOT analyses. (a). Change in telomere length measured using qPCR (aTL). (b). Change in telomere length estimated using DNA methylation (DNAmTL). Change‐scores are quantified as the difference in TL values at the 12‐month and 24‐month follow‐up assessments relative to baseline values (e.g., ΔTL_12_ = TL_BL_−TL_12_; ΔTL_24_ = TL_BL_−TL_24_). Error bars represent standard error of the mean. There is no standard error for baseline assessments as change from baseline is zero. Data points are offset slightly for ease of visualization. Group assignment to “Low CR” and “High CR” are based upon first stage instrumental variables (IV) regression predicting %CR from intervention group status, baseline TL, and baseline demographics as described in Methods. For visualization, assignment to “Low CR” and “High CR” is based on participants projected to have greater or less than 10% CR irrespective of intervention group.

Although the AL group was instructed to continue their normal diets and body weight did not change significantly, AL participants exhibited a wide range of caloric change during the study (i.e., 18% decrease to 21% increase). To explore the general impact of CR on TL dynamics, we estimated the impact of 20% CR on TL in both the CR and AL groups with effect of the treatment on the treated (TOT) analyses using instrumental variable regression (Table 2, Panel C). CR significantly increased DNAmTL attrition between baseline and the 12‐month time point (*p* = 0.031). Attrition in aTL was also greater between baseline and 12 months, reaching statistical significance in sensitivity analyses controlling for covariate effects of oxidative stress and inflammation (*p* = 0.042, Table S13). By contrast, covariate control for immune cell proportions ablated CR effects on aTL during both phases (Table S13). A visual representation of TL change predicted by instrumental variable regression utilized in TOT models is shown in Figure 3.

## 3 DISCUSSION

We investigated the impact of 2 years of moderate CR on TL among healthy adults in the CALERIE™ 2 trial and observed mixed results. ITT models showed trends toward *faster* TL attrition during the weight loss phase (i.e., first year of CALERIE™). Accounting for adherence differences (TOT analyses) (Bang & Davis, 2007; Sussman & Hayward, 2010), we estimated that a 20% caloric reduction would be associated with a significant DNAmTL attrition between baseline and 12 month follow up, a finding that was also reproduced for aTL measurements in sensitivity analyses adding covariate control for processes of inflammation and oxidative stress. Whether CR effects on TL attrition persist or operate in a transient manner is unclear, as differences in TL attrition between CR and AL groups were reduced or resolved by 24 months. Indeed, CR participants exhibited significantly less aTL attrition between 12 and 24 months (i.e., weight maintenance phase). These patterns were reproduced in dose–response analyses within those in the CR group with higher adherence (> = 10% CR). Combined with maintained rates of attrition in the AL group, this resulted in no group differences in aTL or DNAmTL attrition across the full two‐year intervention.

Although CR modulates processes related to telomere stability (i.e., reduced inflammation and oxidative stress), direct evidence for the effect of CR on TL in humans is mixed. Previous work in the PREDIMED‐Plus study reported null effects of CR on TL (Fernandez de la Puente et al., 2021), as did a meta‐analysis of five RCTs covering nine different diets, although some studies reported significantly slower TL attrition (Perez et al., 2017). By contrast, cross‐sectional analyses of long‐term CR practitioners (~10 years) were reported as having shorter average TL compared to demographically matched controls (Tomiyama et al., 2017). One possible reason for the mixed findings in prior studies is difficulty distinguishing effects of CR from effects of obesity‐related weight loss. Obesity and elevated adiposity contribute to a pro‐inflammatory state that increases oxidative stress (Bondia‐Pons et al., 2012), and meta‐analyses have provided evidence for relationships between obesity, increased BMI, and shorter TL (Gielen et al., 2018; Mundstock et al., 2015). By reducing proinflammatory adipokines, weight loss interventions can promote telomere stability, with several studies showing telomere lengthening in response to dietary, exercise, or surgical induced weight loss over periods ranging from 2 months to 10 years (Welendorf et al., 2019).

The design of CALERIE™ Phase 2 addresses this difficulty; all participants were non‐obese, and weight loss occurred predominantly during the first year of the CR intervention, while the second year was characterized by weight maintenance. Specifically, the majority of weight loss in CALERIE™ occurred by week 60, after which diet and CR recommendations were adjusted to maintain weight (Dorling et al., 2020). Our results suggest CR‐mediated weight loss can induce accelerated TL attrition in the first year, possibly due to decreased prioritization of telomere maintenance associated with metabolic scarcity, in line with the metabolic telomere attrition hypothesis (Casagrande & Hau, 2019). In contrast, results reveal the opposite pattern during the weight‐maintenance phase of the intervention in year 2; TL attrition in the CR group *slowed* relative to the AL group during months 12–24. This is similar to patterns observed in the smaller SHINE trial, where average TL for weight loss maintainers decreased in the first 3 months of the intervention, but then increased between 3 months and 1 year (Mason et al., 2018). Thus, TL may exhibit a hormesis‐like response to CR wherein the stress of weight loss that accompanies early‐phase CR accelerates TL attrition, which is thereafter mitigated or ablated altogether as new homeostatic norms are established.

We acknowledge limitations. There is no gold standard of telomere measurement applicable for population‐based studies. This investigation included two distinct approaches toward TL quantification, direct measurement of aTL using qPCR and indirect estimation of TL from DNA methylation data (DNAmTL). The moderate agreement (*r* = 0.29) between the two measurements is in line with previous reports (Doherty et al., 2023; Hastings et al., 2022), as are cross‐sectional per year TL attrition rates (Ye et al., 2023) and differences between White and Black Americans (Hansen et al., 2016; Hunt et al., 2008). The capacity to detect change in TL is dependent on measurement precision, follow‐up duration, and the number of longitudinal assessments. The DNA extraction approached utilized by CALERIE™ (i.e., Qiagen Puregene) has also been shown to increase the variability in qPCR‐based method of TL assessments (Lin et al., 2022). Although we took care to correct aTL measurements using metrics of DNA integrity (Wolf et al., 2024), it remains possible that pre‐analytical factors could have contributed to variability in aTL measurements and change scores. Average within‐person changes in aTL across 2 years, 510 bp, is larger than would be expected based upon chronological age associated differences in aTL observed in linear models (87 bp/year). This difference is partly attributable to intervention effects on aTL but does not preclude the possibility of measurement error contributing to observed findings. In particular, the reversed directionality of aTL attrition in the CR group between the weight loss and weight maintenance phases could also result from regression to the mean. Even so, the strong reliability of aTL measurements (ICC >0.80), consistency of attrition in the AL group, and use of three time points decreases the likelihood that effects are artifacts resulting from measurement error or regression to the mean (Steenstrup et al., 2013).

DNAmTL is not a perfect proxy for TL, reflecting an algorithm of CpG sites trained to predict TL measured using southern blot. Sex differences in DNAmTL presented in the direction opposite expectations, with females exhibiting shorter DNAmTL at all time points. This could be attributed to bias in the predominately female CALERIE™ sample, however, DNAmTL was constructed in a similarly skewed dataset (75% female), and effects in test datasets were in the expected direction (i.e., shorter DNAmTL in men) (Lu et al., 2019). Recent evidence has highlighted how DNAmTL measures trained in disparate datasets (Doherty et al., 2023) can exhibit similar concordance with qPCR‐based measurements of TL despite having low overlap in their CpG composition (<2%), suggesting a broader set of global CpGs related to TL than is comprised in a single DNAmTL algorithm. Given that effects of CR were differential between DNAmTL and aTL measurements, it is possible that the DNAmTL measure utilized in our work registered telomere dynamics especially sensitive to CR (via unique CpGs) that may not replicate to other DNA‐methylation based predictors of TL. TL is not a static measure, representing an average across chromosome ends and various cell types. Although we took care to control for differences in immune cell distribution, it is possible that CR‐mediated effects could translate differentially across one or many chromosomes. This will be a future avenue of research as approaches toward chromosome‐specific TL measurement continue to develop.

Cohen's f^2^ effect sizes for significant group differences observed using CALERIE™ multiple regression frameworks were small for aTL (*f* ^2^_12 months_ = 0.027; *f* ^2^_Maintenance_ = 0.036) and very small for DNAmTL (*f* ^2^_12 months_ = 0.001). Power analyses suggest the study sample size was insufficient to reliably detect effects of this size (*β*_aTL_, _12 months_ = 0.54; *β*_aTL_, _Maintenance_ = 0.62; *β*_DNAmTL_, _12 months_ = 0.07). Thus, results should be taken as exploratory, and effects of CR on TL observed in very healthy, predominately white, majority female participants without obesity may not extend to a more heterogenous general population.

In conclusion, we observed mixed evidence for effects of the CALERIE™ intervention on TL attrition. Intervention group status was not significantly associated with TL attrition across the first year, although there were trends toward increased attrition in the CR group for both TL measurement approaches. Using TOT analysis, which estimated impacts of CR across both intervention groups, increased CR was associated with accelerated DNAmTL attrition between baseline and the 12‐month follow‐up. By contrast, both CR group status and increased CR were associated with slower aTL attrition over the second year of weight maintenance. No differences were observed for either measure when considering TL change across the full study duration from baseline to 24 months, leaving it unclear whether CR‐related impacts reflect long‐term detriments to telomere fidelity, a hormesis‐like adaptation to decreased energy availability, or regression to the mean induced by measurement error.

Although there is a mechanistic basis for CR‐induced telomere attrition in humans, gerontological research suggests different measurement approaches could capture different aspects of the aging process (Belsky et al., 2018; Hastings et al., 2019). Previous findings on the impact of CR on biological aging in CALERIE™ have reflected these differences; CR was associated with a slowed pace of biological aging as measured by blood‐chemistry and organ‐function tests (Belsky et al., 2017), and the DunedinPACE pace‐of‐aging epigenetic clock (Waziry et al., 2023). However, other epigenetic clocks did not respond to CALERIE™ intervention. The lack of consistent findings in our results, combined with underpowered detection of small effect sizes, suggest intervention impacts may be too subtle for detection with high‐throughput measures of TL. Unraveling these trends will be a focus of future work in the CALERIE™ Legacy study and the longer‐term trials of 5 years of calorie restriction and time‐restricted eating.

## 4 METHODS

### 4.1 Study design and participants

CALERIE™ Phase‐2, was a multicenter, randomized controlled trial conducted at three clinical centers in the USA (Rochon et al., 2011). CALERIE™ 2 aimed to evaluate the time‐course effects of 25% CR (i.e., intake 25% below the individual's baseline level) over a 2‐year period in healthy adults (men aged 21–50 y, premenopausal women aged 21–47 y) with BMI in the normal weight or slightly overweight range (BMI 22.0–27.9 kg/m^2^). The study protocol (NCT00427193) was approved by Institutional Review Boards at three clinical centers (Washington University School of Medicine, St Louis, MO, USA; Pennington Biomedical Research Center, Baton Rouge, LA, USA; Tufts University, Boston, MA, USA) and the coordinating center at Duke University (Durham, NC, USA). All study participants provided written informed consent. Non‐genomic data were obtained from the CALERIE™ 2 Biorepository (https://calerie.duke.edu/database‐documentation/data‐contents ).

### 4.2 Procedures

Study procedures have been published previously (Kraus et al., 2019; Racette et al., 2012; Ravussin et al., 2015) and are described here in brief. After baseline testing, participants were randomized using a 2:1 ratio in favor of CR or to an AL control group. A permuted block randomization technique was used, and randomization was stratified by site, sex, and BMI. Using mathematical models generated by CALERIE™ Phase I studies, participants in the CR group were prescribed weight loss curves to promote 25% restriction in calorie intake (Martin et al., 2022). Participants were provided three meals per day, and three different eating plans, for 27 days to familiarize themselves with portion sizes and meal types for the prescribed reduced calorie intake. Participants received instruction on the essentials of CR and attended intensive group and individual behavioral counselling sessions once per week over the first 24 weeks of the intervention. Responsiveness to the CR intervention was assessed in real time by the degree to which individual weight change followed a predicted weight loss trajectory (15.5% weight loss at 1 year followed by weight maintenance). Participants assigned to the AL group continued their regular diets; they had quarterly contact with study investigators to complete assessments but received no dietary intervention or counselling.

### 4.3 Quantification of %CR

Mean %CR was calculated at each time point as percent decrease in energy intake relative to baseline energy requirements using the equation: $\%{\mathrm{CR}}_{\text{Mean}}=\frac{1-{\mathrm{EI}}_{\text{Mean}}}{{\mathrm{EI}}_{\mathrm{BL}}}\times 100$, where EI_BL_ is the Total Energy Expenditure (TEE) at pre‐intervention baseline and EI_Mean_ is the weighted average of TEE across all follow‐up visits up through the assessment at which %CR was calculated (Racette et al., 2012). TEE was measured by the doubly labeled water method during two consecutive 2‐week periods at baseline and for 2 weeks at each follow‐up assessment. TEE for %CR at 12‐months was calculated using the average of measurements at 6 and 12 months. TEE for %CR at 24 months was calculated as the average of measurements 6, 12, 18, and 24 months.

### 4.4 TL measurement

We used two approaches for TL measurement, direct assessment of aTL using qPCR (O'Callaghan & Fenech, 2011), and indirectestimation of DNAmTL using the DNA methylation EPIC v1 array  (Lu et al., 2019). Methods for aTL and DNAmTL measurement are described in detail in Appendix S1. Further methodological details for qPCR assay to measure aTL are provided in Table S15 in line with the Telomere Research Network Reporting Guidelines.

### 4.5 Oxidative stress and inflammation

We accounted for processes of oxidative stress using a principal component (PC1) accounting for 73.39% of variance in four F2‐isoprostanes (Table S16). F2‐isoprostanes were measured in urine using liquid chromatography–tandem mass spectrometry and adjusted for urinary creatinine as previously described (Il'yasova et al., 2018). Inflammatory biomarkers were measured using immunoassays in plasma from fasting blood samples as previously described (Meydani et al., 2016). We accounted for processes of inflammation using a PC1 accounting for 23.74% of variance in seven inflammatory markers (CRP, ICAM1, IL‐6, IL‐8, Leptin, MCP1, & TNF‐α; Table S17 and S18).

### 4.6 Statistical analysis

Our analytic approach followed models used in prior analyses of the CALERIE™ Trial (Kraus et al., 2019; Waziry et al., 2023). We characterized associations between TL measurements assessed using qPCR (aTL) and DNA methylation (DNAmTL) across all three time points and external validity metrics (i.e., age, sex, race, BMI) using general linear models with random effects at the participant level to account for correlation of repeated TL measurements within individuals. General linear models took the form: $\mathrm{TL}=\beta_{0}+\sum \beta_{i}x_{i}+\gamma +\varepsilon$, where β_0_ is the intercept, β_i_ are effects for external validity metrics, γ is the random individual effect and ε is the error term.

We tested the hypothesis that CR slows TL attrition by computing TL change‐scores as the difference in values at the 12‐month and 24‐month assessments relative to baseline values (e.g., ΔTL_12_ = TL_BL_−TL_12_; ΔTL_24_ = TL_BL_−TL_24_). We tested for effects specific to the weight maintenance phase by computing TL change scores as the difference in values at 24 months relative to 12 months (i.e., ΔTL_Maintenance_ = TL_24_−TL_12_). Change scores closer to zero reflect less TL attrition, and positive change scores reflect an increase in TL over time. We analyzed these change scores using two complementary approaches: (1) Intent‐to‐Treat (ITT) analysis and (2) Effect‐of‐Treatment‐on‐the‐Treated (TOT) analysis. All analyses were conducted in R (v 4.3.1). Homogeneity of variances was assessed using Levene's Test. Normality of change scores across groups failed initial evaluation by the Shapiro–Wilk test. After the removal of a small number (*N* = 17, 4.8%) of aTL change scores identified as outliers using the *boxplot* function in R (AL_12_ = 4, AL_24=_2, CR_12_ = 6, CR_24_ = 5), the retained data met all model assumptions.

ITT analyses tested the effect of randomization to AL versus CR on TL change‐scores under mixed models, following the approach used in past CALERIE™ analyses. Models include terms for treatment condition (AL or CR; β_1_), follow‐up time (β_2_), an interaction term modeling heterogeneity in the treatment effect between the 12‐ and 24‐month time points (β_3_), pre‐treatment covariates (chronological age, sex, race, BMI and study site; β_i_), and baseline TL values to control for regression‐to‐the‐mean effects (β_4_): $∆\mathrm{TL}=\beta_{0}+\beta_{1}\text{Group}+\beta_{2}\text{Time}+\beta_{3}\text{Group}\times \text{Time}+\beta_{4}{\mathrm{TL}}_{\text{Base}}+\sum \beta_{i}x_{i}+\gamma +\varepsilon$. Models were fit using *"lmer"*. Intervention effects at 12 and 24 months were estimated using *margins*.

TOT analysis tested the effect of the CR intervention on TL change scores using IV regression implemented using a two‐stage least squares approach (Sussman & Hayward, 2010). Concomitance of weight‐loss and CR adherence was imperfect in CALERIE™; although participants remained within individually bounded weight‐loss trajectories, these zones of adherence did not correspond to exactly 25% CR (Martin et al., 2022). As a result, average CR achieved in the treatment group was roughly ½ the prescribed dose of 25% (i.e., mean = 11.9%, SE = 0.7%). Under conditions of less than 100% adherence, traditional ITT analysis can result in a biased estimate of the treatment effect, and an IV estimator can provide a complement (Bang & Davis, 2007; Sussman & Hayward, 2010). The IV approach involved three related regressions that sequentially (i) identify factors significantly associated with %CR, representing factors influencing adherence, (ii) use these factors to model %CR, and (iii) extract fitted values for %CR to predict change in TL. Separate models are fit for scores reflecting change in TL from baseline to 12 months (i.e., weight loss phase: N_AL_ = 59, N_CR_ = 111), baseline to 24 months (N_AL_ = 64, N_CR_ = 107), and 12 months–24 months (i.e., weight maintenance phase; N_AL_ = 57, N_CR_ = 101). Full details on TOT model construction are provided in Appendix S1. Models are fit using the “*ivreg*” function. Because few individuals attained the prescribed 25% CR, we estimated the impact of 20% CR on TL attrition.

We conducted two sets of sensitivity analyses. First, we tested sensitivity of TOT models to processes of oxidative stress and inflammation using change in PC factor scores. We also tested sensitivity of TOT models to changes in white blood cell proportions (lymphocytes, monocytes, and neutrophils) in two different models by including terms for (i) cell count estimates derived from complete blood count (CBC) or (ii) cell count estimates from DNA methylation data using the Houseman Equation via the *minfi* and *FlowSorted.Blood.EPIC* R packages (Aryee et al., 2014; Salas et al., 2018).

### 4.7 Power analyses

We estimated the power to detect small and medium effects of CR on TL change scores in ITT and TOT models as Cohen's f^2^ values derived from full versus reduced models in multiple regression (Maxwell et al., 2017). In this approach, we operationalized Cohen's f^2^ values as effects detectable in a reduced model controlling for baseline covariates versus a full model with the added effect of interest (i.e., intervention group status or %CR). Recommended criterion for small (*f* ^2^ = 0.02) and medium (*f* ^2^ = 0.15) effects were scaled for measurement reliability by multiplying effects by each measurement's intraclass correlation coefficient (ICC_aTL_ = 0.834; ICC_DNAmTL_ = 0.994) prior to performing power analyses (Williams & Zimmerman, 1989). Since measurement error of change scores is instantiated twice, first on baseline measurements and again on follow‐up assessments, ICC values were not square rooted during effect scaling. Power to detect effects in a full model of seven predictors versus a reduced model of six predictors were generated using the "*wp.regression*" command of the *WebPower* R package.

Results indicate that the study had sufficient power to detect medium effects on aTL (*β* = 0.99) and DNAmTL (*β* = 0.99) but was underpowered to detect small effects on either measure (*β*_aTL_ = 0.39; *β*_DNAmTL_ = 0.45).

## AUTHOR CONTRIBUTIONS

All authors participated in different aspects of the study. I.S. designed and supervised the study. W.J.H. generated qPCR aTL data, performed statistical analysis, and wrote the original manuscript. D.W.B., C.P.R., M.S.K., and J.L.M. supported bioinformatics work and generation of DNAmTL data. Q.Y., S.E.W., D.W.B., and C.P.R. supported computational and statistical analysis design. S.K.D., C.K.M., S.B.R., L.M.R., K.M.R., W.E.K critically reviewed the manuscript and provided important intellectual content. All authors reviewed the manuscript and approved for submission.

## CONFLICT OF INTEREST STATEMENT

DWB is listed as an inventor of DunedinPACE, a Duke University and University of Otago invention licensed to TruDiagnostic.

## Supporting information


Appendix S1.

## Data Availability

All data and materials that support the findings of this study are available within the manuscript and supplemental information. Telomere length data were deposited in CALERIE™ bio repository that they can be obtained by request from the CALERIE™ bio repository. Any information required to reanalyze the data reported in this paper is available from the lead contact upon request.
