# Supplementary material for: Effect of long‐term caloric restriction on telomere length in healthy adults: CALERIE™ 2 trial analysis
Source: Aging Cell. 2024 Mar 19;23(6):e14149. doi: 10.1111/acel.14149 (PMC11296136; doi:10.1111/acel.14149)
Supplement: Supplementary file 1 — Appendix S1. [file ACEL-23-e14149-s001.docx]

**SUPPLEMENTAL METHODS**

## Absolute telomere length (aTL)

DNA was extracted from blood samples using Puregene kits (Qiagen). DNA was shipped from the CALERIE™ Biorepository at the University of Vermont to Pennsylvania State University for aTL assays following a qPCR method originally developed by O’Callaghan and Fenech (O'Callaghan & Fenech, 2011). DNA quality was assessed using the DNA Integrity Number (DIN) generated by the Agilent 4150 TapeStation. The average DIN for all samples was 9.40, indicating intact, high-quality DNA.

All qPCR assays were loaded using a Qiagility robotic pipette (Qiagen) and run on a Rotor-Gene Q thermocycler connected to an uninterruptible power source (CyberPower), which has been shown to decrease variability in TL measured via qPCR (Hastings et al., 2020). Each qPCR assay was comprised of two runs, one quantifying telomere content, and a second run quantifying genome copy number using the single copy gene *IFNB1*. Each run hosted triplicate reactions of 22 samples, 6 standards, 5 controls, and 1 no-template control (singlet) on 100 well disks.

Telomere standards were constructed using an 84 bp double-stranded oligomer comprised of 16 copies of canonical telomere repeat. *IFNB1* standards were constructed using an 83 bp double-stranded oligomer corresponding to the genomic region of *IFNB1* flanked by *IFNB1* primers. Estimates of kb telomeric DNA and genome copy number, in triplicate, were calculated in accordance with a sample’s alignment to each standard curve. Sample-level aTL values were calculated using the formula: $aTL=\frac{Estimated kb Telomeric DNA}{Estimated Genome Copy Number\times92}$.

To control for inter-assay variability, five control samples were included on every qPCR assay. Four controls were randomly selected from within the sample and the final control sample was comprised of DNA extracted from the Jurkat cell line (ThermoFisher), which is known to have short telomere length (<6kb). The average inter-assay CV across all 32 assays was 7.54%. Additional correction was performed by residualizing aTL measurements onto metrics of DNA degradation and double stranded DNA concentration derived from the Agilent 4150 TapeStation (Wolf et al., 2024). To preserve the original distribution, we then added the model intercept to residual values. Corrected aTL measurements were highly correlated with original values (r=0.978). Measurement reproducibility was assessed as the intraclass correlation coefficient (ICC) across a total of 88 samples with duplicate aTL values. The ICC for aTL estimates across the cohort was 0.834, indicating good reliability. Full details on qPCR assays for aTL, including reaction mix composition and sequences for primers and standards, are summarized in **Supplemental Table S14** in accordance with guidelines recommended by the Telomere Research Network (doi: 10.31219/osf.io/9pzst). Following quality control assessment aTL measurements were available for n=571 samples (baseline n=206, 12-months n=185, 24-months n=180).

## DNA methylation telomere length (DNAmTL)

DNA methylation data were generated at the University of British Columbia and processed by the Genomic Analysis and Bioinformatics Shared Resource at Duke University (Ramaker et al., 2022). Illumina Infinium Methylation EPIC BeadChip arrays were used to assay genome-wide DNA methylation data from banked DNA samples extracted from whole blood collected at the baseline, 12- and 24-month follow-ups. The EPIC array quantifies DNAm levels at >850,000 CpG sites across all known genes, regions, and key regulatory regions. Briefly, 750ng of extracted DNA samples were bisulfite converted using the EZ DNA Methylation kit (Zymo Research, Irvine, CA), and 160ng of the converted DNA were used as input for the EPIC arrays (Illumina, San Diego, CA). EPIC arrays were processed according to the manufacturer’s instructions and scanned using the Illumina iScan platform. To the extent possible, baseline, 12-month, and 24-month samples from the same individual were processed in the same array batch and on the same Beadchip to minimize batch effects; CR treatment and AL control participants were included on all chips. Quality control and normalization analyses were performed using the methylumi (v 3.13) (Davis, 2021) Bioconductor (v 2.46.0) (Huber et al., 2015) package for the R statistical programming environment (v 3.6.3). Probes were considered missing in a sample if they had detection p-values>0.05 and were excluded from the analysis if they were missing in >5% of sample. Normalization to eliminate systematic dye bias in 2-channel probes was carried out using the methylumi default method. Following quality control and normalization, DNA methylation data for 828,613 CpGs were available for n=595 samples (baseline n=214; 12-months n=193; 24-months n=188).

We analyzed a version of the DNAmTL constructed from principal components, which have superior technical reliability as compared to the original version of this measure (Higgins-Chen et al., 2022). Additional batch correction was performed by residualizing DNAmTL measurements onto principal components estimated from array control-probe beta values (Lehne et al., 2015). To preserve the original distribution, we then added the model intercept to residual values. Batch corrected DNAmTL measurements were highly correlated with original values (r=0.929). Cell count estimation was performed using the Houseman Equation via the *minfi* and *FlowSorted.Blood.EPIC* R packages (Aryee et al., 2014; Salas et al., 2018).

**Effect of Treatment on the Treated (TOT) Analysis**

TOT analysis tested the effect of the CR intervention on TL change scores using instrumental variables (IV) regression implemented using a two-stage least squares approach (Sussman & Hayward, 2010). Concomitance of weight-loss and CR adherence was imperfect in CALERIE™; although participants remained within individually bounded weight-loss trajectories, these zones of adherence did not correspond to exactly 25% CR (Martin et al., 2022). As a result, average CR achieved in the treatment group was roughly ½ the prescribed dose of 25% (i.e., mean= 11.9%, SE=0.7%). Under conditions of non-adherence, traditional ITT analysis can result in a biased estimate of the treatment effect and an IV estimator can provide a complement (Bang & Davis, 2007; Sussman & Hayward, 2010). The IV approach involved three related regressions.

The first regression models CR-treatment dose at 12- and 24-month follow-ups (%CR_t_) as a function of randomization group (CR vs AL), pre-treatment characteristics (chronological age, sex, race, BMI, study site, and baseline TL), and two-way interactions among pretreatment covariates (*interactions between race and study site/sex were omitted due to insufficient site- and sex-specific variation in race to fit models*). Variables identified as significant predictors of %CR are then used in a second, reduced regression to model %CR for the total sample by including their interaction with CR group status and baseline TL.

In the first stage, we identified participant race as a significant predictor of %CR in this sample. Thus, we parameterized our IV regression by including the instrument of intervention group (AL or CR; β_1_), an interaction between group and race (β_2_), an interaction between race and baseline TL (β_3_), and individual effects of all pre-treatment covariates including race and baseline TL (β_i_): ${\%CR}_{t}=\beta_{0}+\beta_{1}Group+B_{2}Group*Race+\beta_{3}Group*{TL}_{Base}+\sum\beta_{i}x_{i}+\varepsilon$.

Fitted values for %CR are extracted and used in a final regression model to predict change in TL: $\Delta TL = \beta_{0}+\beta_{1}{\%CR}_{t}+\sum\beta_{i}x_{i}+\varepsilon$, where %CR_t_ is the value estimated from the first equation, β_0_ is the intercept, β_i_ are the effect of pretreatment covariates, ε is the error, and β_1_ is our effect of interest. Separate models are fit for scores reflecting change in each TL measurement (aTL & DNAmTL) from baseline to 12 months (i.e., weight loss phase; N_AL_=58, N_CR_=112), baseline to 12 months (N_AL_=64, N_CR_=109), and 12 months to 24 months (i.e., weight maintenance phase; N_AL_=56, N_CR_=103). Models fit for maintenance effects, i.e., ΔTL_Maintenance_ =TL_24_ – TL_12_, were parameterized using %CR estimated at 24 months, i.e., %CR_24_. Models are fit using the ‘*ivreg*’ function in R (v 4.3.1). Because few individuals attained the prescribed 25% CR, we estimated the incremental impact of 20% increase in CR on TL attrition.

**SUPPLEMENTAL TABLES AND FIGURES**

**Table S1: Baseline characteristics of analytical sample of individuals with data for both TL measures at baseline and at least one follow-up assessment.** p-values illustrate results of student’s t (*age*) or chi-square tests of differences between AL and CR groups.

|  | **Analysis Sample** | | **Ad Libitum** | | **Caloric Restriction** | |  |
| --- | --- | --- | --- | --- | --- | --- | --- |
|  | **Mean** | **SD** | **Mean** | **SD** | **Mean** | **SD** | **p** |
| **Baseline Age (years)** | 38.58 | 7.02 | 38.49 | 7.02 | 38.62 | 7.05 | 0.902 |
| **Sex** | **N** | **%** | **N** | **%** | **N** | **%** |  |
| *Women* | 122 | 69.7% | 43 | 68.3% | 79 | 70.5% | 0.886 |
| *Men* | 53 | 30.3% | 20 | 31.7% | 33 | 29.5% |  |
| **Race/ethnic identity** |  |  |  |  |  |  |  |
| *White* | 135 | 77.1% | 47 | 74.6% | 88 | 78.6% | 0.351 |
| *Black* | 20 | 11.4% | 10 | 15.9% | 10 | 8.9% |  |
| *Other* | 20 | 11.4% | 6 | 9.5% | 14 | 12.5% |  |
| **Study Site** |  |  |  |  |  |  |  |
| *A* | 54 | 30.9% | 19 | 30.2% | 35 | 31.3% | 0.920 |
| *B* | 66 | 37.7% | 25 | 39.7% | 41 | 36.6% |  |
| *C* | 55 | 31.4% | 19 | 30.2% | 36 | 32.1% |  |
| **BMI Stratum** |  |  |  |  |  |  |  |
| *Lean* | 81 | 46.3% | 29 | 46.0% | 52 | 46.4% | 0.999 |
| *Overweight* | 94 | 53.7% | 34 | 54.0% | 60 | 53.6% |  |

**Table S2: Tests for Difference in baseline characteristics between analytical sample (individuals with data for both TL measures at baseline and at least one follow up assessment) and remaining CALERIE™ participants.** p-values illustrate results of student’s t (*age*) or chi-square tests of differences between analysis sample and participants without TL data

|  | **Analysis Sample** | | **Remaining Participants** | |  |
| --- | --- | --- | --- | --- | --- |
|  | **Mean** | **SD** | **Mean** | **SD** | **p** |
| **Baseline Age (years)** | 38.42 | 7.09 | 35.93 | 7.73 | 0.103 |
| **Intervention Group** | **N** | **%** | **N** | **%** |  |
| *AL* | 63 | 36.1% | 8 | 36.10% | 0.503 |
| *CR* | 112 | 63.9% | 21 | 63.90% |  |
| **Sex** |  |  |  |  |  |
| *Women* | 122 | 69.7% | 21 | 72.4% | 0.940 |
| *Men* | 53 | 30.3% | 8 | 27.6% |  |
| **Race/ethnic identity** |  |  |  |  |  |
| *White* | 135 | 77.1% | 24 | 82.8% | 0.741 |
| *Black* | 20 | 11.4% | 3 | 10.3% |  |
| *Other* | 20 | 11.4% | 2 | 6.9% |  |
| **Study Site** |  |  |  |  |  |
| *A* | 54 | 30.9% | 8 | 27.6% | 0.916 |
| *B* | 66 | 37.7% | 12 | 41.4% |  |
| *C* | 55 | 31.4% | 9 | 31.0% |  |
| **BMI Stratum** |  |  |  |  |  |
| *Lean* | 81 | 46.3% | 18 | 62.1% | 0.169 |
| *Overweight* | 94 | 53.7% | 11 | 37.9% |  |

**Table S3: Correlation of TL measures and chronological age.**

Panel A: correlation of baseline measurements only. Panel B: correlation of measurements at 12 months. Panel C: correlation of measurements at 24 months. *p<0.01; **p<0.001; ***p<0.0001.

| Panel A: Baseline assessment | | |
| --- | --- | --- |
|  | **Age** | **aTL** |
| **Age** | - |  |
| **aTL** | -0.26** | - |
| **DNAmTL** | -0.74*** | 0.29*** |
| Panel B: 12-month assessment | | |
|  | **Age** | **aTL** |
| **Age** | - |  |
| **aTL** | -0.24** | - |
| **DNAmTL** | -0.70*** | 0.27** |
| Panel C: 24-month assessment | | |
|  | **Age** | **aTL** |
| **Age** | - |  |
| **aTL** | -0.26* | - |
| **DNAmTL** | -0.71*** | 0.26** |

**Table S4: TL measurements for the full sample and each intervention group at baseline and follow up**

**Top Panel**: Summary statistics of aTL measurements generated using qPCR. **Bottom Panel**: Summary statistics of DNAmTL measurements generated using DNA methylation. Values shown are Mean (Standard Deviation). P-values report results of t-test for differences between AL and CR groups at each time point**.**

| aTL | | | | |
| --- | --- | --- | --- | --- |
|  | **Full Sample** | **AL** | **CR** | **p** |
| Baseline | 7.33 (2.27) | 7.42 (2.27) | 7.26 (1.25) | 0.662 |
| 12 Month | 6.93 (1.93) | 7.18 (2.04) | 6.80 (1.66) | 0.245 |
| 24 Month | 6.82 (1.85) | 6.86 (1.75) | 6.80 (1.92) | 0.837 |
| DNAmTL | | | | |
|  | **Full Sample** | **AL** | **CR** | **p** |
| Baseline | 7.21 (0.17) | 7.20 (0.16) | 7.21 (0.17) | 0.863 |
| 12 Month | 7.20 (0.16) | 7.20 (0.14) | 7.19 (0.17) | 0.692 |
| 24 Month | 7.16 (0.16) | 7.17 (0.15) | 7.16 (0.17) | 0.633 |

**
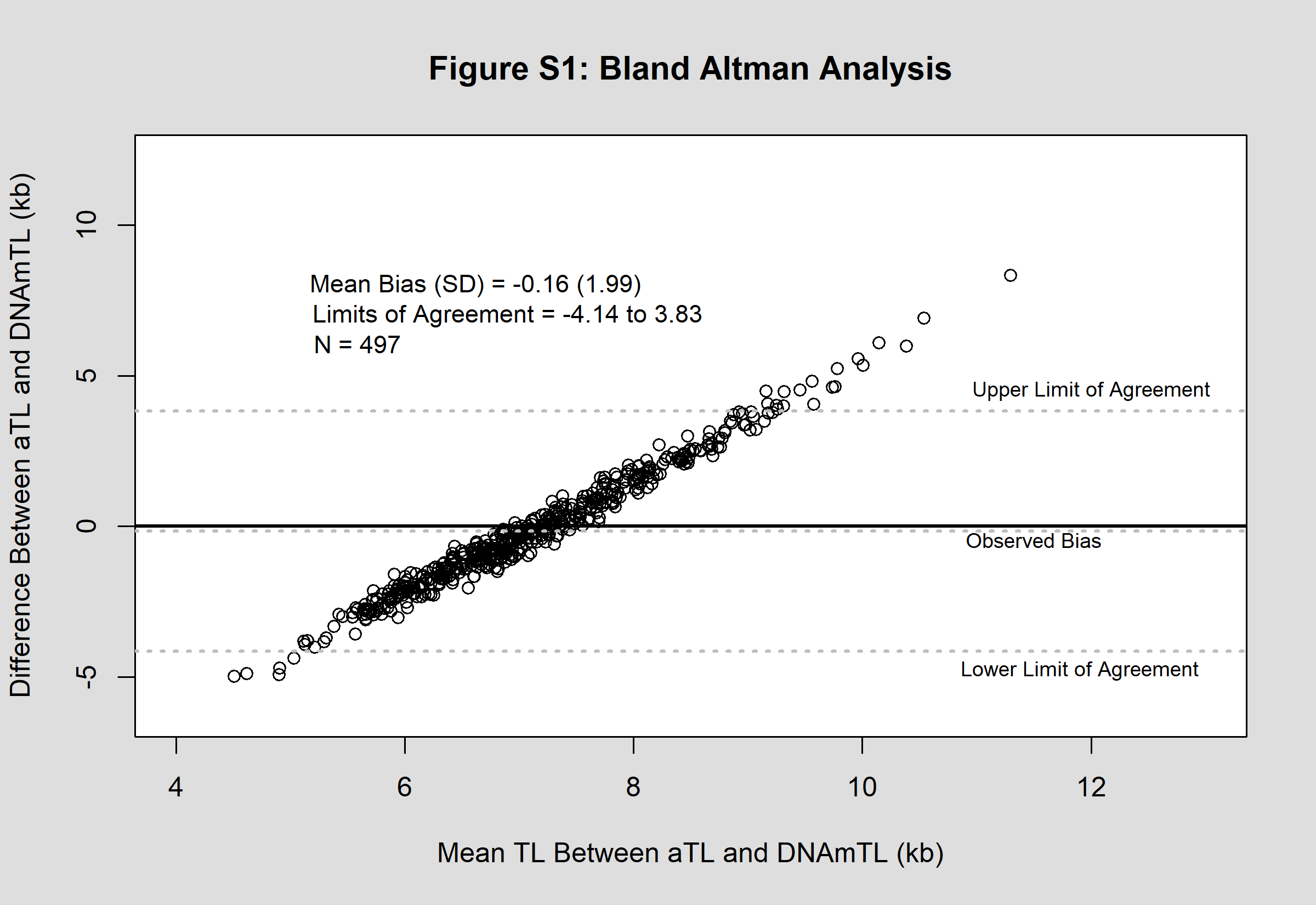
**

**Figure S1: Bland-Altman analysis of aTL and DNAmTL in CALERIE™.** X-axis represents the average of the two measurements. The Y-axis represents the difference between the two measurements. Each point corresponds to one paired comparison. As in previous studies (Hastings et al., 2022), aTL measurements represent a wider distribution of TL values than do DNAmTL measurements, whose distribution is compressed as a result of its derivation from a finite CpG set of changes, each associated with discrete base-pair intervals.

**Table S5**: **Standardized effect sizes for** g**eneral linear models testing associations between TL and sample demographics.** Continuous variables (aTL, DNAmTL, age) were z-scored prior to analyses. Coefficient for age reflects SD difference in TL measures for each SD change in age. Coefficients for BMI, Sex, and Race are SD difference in TL relative to reference groups of Lean BMI, males, and Whites. Significant effects in bold.

|  | **aTL** | | | **DNAmTL** | | |
| --- | --- | --- | --- | --- | --- | --- |
|  | **𝛽** | **[95% CI]** | **p** | **𝛽** | **[95% CI]** | **p** |
| Intercept | -0.16 | [-0.49, 0.16] | 0.322 | 0.11 | [-0.13, 0.36] | 0.361 |
| Age | -0.30 | [-0.43, -0.18] | **<0.001** | -0.74 | [-0.82, -0.66] | **<0.001** |
| Sex | 0.15 | [-0.16, 0.45] | 0.347 | -0.26 | [-0.48, -0.03] | **0.027** |
| BMI: Overweight | -0.05 | [-0.33, 0.22] | 0.704 | 0.07 | [-0.14, 0.27] | 0.526 |
| Race: Black | 0.58 | [0.16, 1.00] | **0.007** | 0.38 | [0.07, 0.69] | **0.019** |
| Race: Other | 0.24 | [-0.18, 0.66] | 0.257 | -0.09 | [-0.41, 0.22] | 0.556 |

**Table S6: General linear models testing associations between TL and sample demographics stratified by sample occasion.** Coefficient for age reflects kb difference in TL for each one-year increase in chronological. Coefficients for BMI, Sex, and Race are kb difference relative to reference groups of Lean BMI, males, and Whites. Significant effects indicated in bold.

| **BASELINE** | | | | | | |
| --- | --- | --- | --- | --- | --- | --- |
|  | **aTL** | | | **DNAmTL** | | |
|  | **𝛽** | **[95% CI]** | **p** | **𝛽** | **[95% CI]** | **p** |
| Intercept | 9.84 | [7.81, 11.88] | **<0.001** | 7.92 | [7.82, 8.03] | **<0.001** |
| Age | -0.08 | [-0.12, -0.03] | **0.001** | -0.02 | [-0.02, -0.02] | **<0.001** |
| Sex | 0.51 | [-0.24, 1.26] | 0.182 | -0.04 | [-0.08, 0.00] | **0.040** |
| BMI: Overweight | -0.05 | [-0.73, 0.63] | 0.895 | 0.01 | [-0.03, 0.04] | 0.770 |
| Race: Black | 0.83 | [-0.21, 1.86] | 0.118 | 0.08 | [0.03, 0.13] | **0.003** |
| Race: Other | 0.36 | [-0.67, 1.40] | 0.494 | 0.00 | [-0.05, 0.05] | 0.944 |
| **12 MONTHS** | | | | | | |
|  | **aTL** | | | **DNAmTL** | | |
|  | **𝛽** | **[95% CI]** | **p** | **𝛽** | **[95% CI]** | **p** |
| Intercept | 8.84 | [7.02, 10.66] | **<0.001** | 7.85 | [0.05, 7.97] | **<0.001** |
| Age | -0.06 | [-0.10, -0.02] | **0.004** | -0.02 | [0.00, -0.01] | **<0.001** |
| Sex | 0.40 | [-0.25, 1.04] | 0.229 | -0.04 | [0.02, 0.00] | 0.086 |
| BMI: Overweight | -0.09 | [-0.69, 0.50] | 0.758 | 0.02 | [0.02, 0.05] | 0.361 |
| Race: Black | 1.51 | [0.60, 2.41] | **0.001** | 0.05 | [0.03, 0.11] | 0.067 |
| Race: Other | 0.63 | [-0.26, 1.51] | 0.169 | -0.04 | [0.03, 0.02] | 0.198 |
|  |  |  |  |  |  |  |
| **24 MONTHS** | | | | | | |
|  | **aTL** | | | **DNAmTL** | | |
|  | **𝛽** | **[95% CI]** | **p** | **𝛽** | **[95% CI]** | **p** |
| Intercept | 8.96 | [7.15, 10.77] | **<0.001** | 7.89 | [7.77, 8.00] | **<0.001** |
| Age | -0.06 | [-0.10, -0.02] | **0.002** | -0.02 | [-0.02, -0.01] | **<0.001** |
| Sex | 0.44 | [-0.19, 1.06] | 0.171 | -0.04 | [-0.08, 0.00] | **0.040** |
| BMI: Overweight | -0.02 | [-0.60, 0.55] | 0.941 | 0.01 | [-0.03, 0.05] | 0.561 |
| Race: Black | 1.20 | [0.37, 2.04] | **0.006** | 0.07 | [0.01, 0.12] | **0.021** |
| Race: Other | 0.62 | [-0.28, 1.51] | 0.179 | -0.02 | [-0.07, 0.04] | 0.614 |

**Table S7: General linear models testing associations between TL and sample demographics with additional control for immune cell counts estimated from complete blood count data.** Coefficients reflect kb difference in TL for each one-year increase in chronological age or one percent increase in immune cell percentage**.** Coefficients for BMI, Sex, and Race are kb difference relative to reference groups of Lean BMI, males, and Whites. Significant effects indicated in bold.

|  | **aTL** | | | **DNAmTL** | | |
| --- | --- | --- | --- | --- | --- | --- |
|  | **𝛽** | **[95% CI]** | **p** | **𝛽** | **[95% CI]** | **p** |
| Intercept | 3.12 | [-5.34, 11.59] | 0.470 | 7.94 | [7.59, 8.30] | **<0.001** |
| Age | -0.09 | [-0.12, -0.05] | **<0.001** | -0.02 | [-0.02, -0.02] | **<0.001** |
| Sex | 0.30 | [-0.32, 0.91] | 0.348 | -0.04 | [-0.08, 0.00] | **0.030** |
| BMI | -0.15 | [-0.71, 0.41] | 0.604 | 0.01 | [-0.02, 0.05] | 0.523 |
| Race: Black | 1.18 | [0.32, 2.03] | **0.008** | 0.07 | [0.01, 0.12] | **0.015** |
| Race: Other | 0.52 | [-0.33, 1.37] | 0.234 | -0.02 | [-0.07, 0.04] | 0.559 |
| Lymphocytes | 0.07 | [-0.01, 0.16] | 0.097 | 0.00 | [0.00, 0.00] | 0.799 |
| Monocytes | 0.07 | [-0.05, 0.20] | 0.254 | 0.00 | [0.00, 0.01] | 0.757 |
| Neutrophils | 0.07 | [-0.01, 0.15] | 0.099 | 0.00 | [0.00, 0.00] | 0.787 |

**Table S8: General linear models testing associations between TL and sample demographics with additional control for immune cell counts estimated from DNA methylation data.** Coefficients reflect kb difference in TL for each one-year increase in chronological age or one percent increase in immune cell percentage**.** Coefficients for BMI, Sex, and Race are kb difference relative to reference groups of Lean BMI, males, and Whites. Significant effects indicated in bold.

|  | **aTL** | | | **DNAmTL** | | |
| --- | --- | --- | --- | --- | --- | --- |
|  | **𝛽** | **[95% CI]** | **p** | **𝛽** | **[95% CI]** | **p** |
| Intercept | -4.47 | [-23.81, 14.87] | 0.651 | 7.71 | [6.91, 8.52] | **<0.001** |
| Age | -0.09 | [-0.12, -0.05] | **<0.001** | -0.02 | [-0.02, -0.02] | **<0.001** |
| Sex | 0.28 | [-0.33, 0.90] | 0.368 | -0.04 | [-0.08, -0.01] | **0.019** |
| BMI | -0.14 | [-0.70, 0.43] | 0.637 | 0.01 | [-0.02, 0.04] | 0.611 |
| Race: Black | 1.14 | [0.28, 2.00] | **0.011** | 0.05 | [0.00, 0.11] | **0.041** |
| Race: Other | 0.47 | [-0.39, 1.33] | 0.283 | -0.02 | [-0.07, 0.03] | 0.414 |
| Lymphocytes | 0.14 | [-0.05, 0.33] | 0.138 | 0.00 | [-0.01, 0.01] | 0.499 |
| Monocytes | 0.14 | [-0.06, 0.34] | 0.174 | 0.00 | [-0.01, 0.01] | 0.683 |
| Neutrophils | 0.14 | [-0.04, 0.33] | 0.137 | 0.00 | [-0.01, 0.01] | 0.711 |

**Table S9: Oxidative stress and inflammation principal component scores for the full sample and each intervention group at baseline and follow up**

**Top Panel**: Summary statistics of oxidative stress principal component (*PC1 from Table S15*). **Bottom Panel**: Summary statistics of inflammation principal component (*PC1 from Table S17*). Values shown are Mean (Standard Deviation). P-values report results of t-test for differences between AL and CR groups at each time point**.** Significant effects indicated in bold.

| Inflammation PC1 | | | | |
| --- | --- | --- | --- | --- |
|  | **Full Sample** | **AL** | **CR** | **p** |
| Baseline | 0.61 (1.18) | 0.84 (1.28) | 0.48 (1.11) | 0.057 |
| 12 Month | -0.20 (1.13) | 0.10 (1.09) | -0.36 (1.12) | **0.010** |
| 24 Month | -0.48 (1.25) | -0.26 (1.22) | -0.61 (1.26) | 0.078 |
| Oxidative Stress PC1 | | | | |
|  | **Full Sample** | **AL** | **CR** | **p** |
| Baseline | -0.03 (1.02) | -0.05 (0.99) | -0.01 (1.04) | 0.814 |
| 12 Month | -0.28 (0.71) | -0.16 (0.59) | -0.34 (0.76) | 0.084 |
| 24 Month | -0.22 (0.72) | -0.08 (0.79) | -0.31 (0.67) | 0.054 |

**Table S10: General linear models testing associations between TL and sample demographics with additional control for oxidative stress and inflammation.** Coefficients reflect kb difference in TL for each one-year increase in chronological age or standard deviation increase in oxidative stress or inflammation principal component. Coefficients for BMI, Sex, and Race are kb difference relative to reference groups of Lean BMI, males, and Whites. Significant effects indicated in bold.

|  | **aTL** | | | **DNAmTL** | | |
| --- | --- | --- | --- | --- | --- | --- |
|  | **𝛽** | **[95% CI]** | **p** | **𝛽** | **[95% CI]** | **p** |
| Intercept | 9.89 | [8.26, 11.51] | **<0.001** | 7.90 | [7.81, 8.00] | **<0.001** |
| Age | -0.08 | [-0.12, -0.04] | **<0.001** | -0.02 | [-0.02, -0.02] | **<0.001** |
| Sex | 0.32 | [-0.29, 0.94] | 0.306 | -0.04 | [-0.08, -0.01] | **0.024** |
| BMI | -0.18 | [-0.74, 0.38] | 0.536 | 0.01 | [-0.02, 0.05] | 0.528 |
| Race: Black | 1.13 | [0.28, 1.98] | **0.010** | 0.06 | [0.01, 0.11] | **0.020** |
| Race: Other | 0.48 | [-0.37, 1.33] | 0.270 | -0.02 | [-0.07, 0.03] | 0.510 |
| Oxidative Stress | -0.01 | [-0.18, 0.15] | 0.886 | 0.00 | [-0.01, 0.01] | 0.837 |
| Inflammation | 0.16 | [0.06, 0.25] | **0.001** | 0.00 | [-0.01, 0.00] | 0.328 |

**Table S11: General linear models testing associations between TL and individual inflammatory markers.** Coefficients reflect kb difference in TL for each one-year increase in chronological age or unit increase in biomarker concentration. Coefficients for BMI, Sex, and Race are kb difference relative to reference groups of Lean BMI, males, and Whites. Significant effects indicated in bold.

|  | **aTL** | | | **DNAmTL** | | |
| --- | --- | --- | --- | --- | --- | --- |
|  | **𝛽** | **[95% CI]** | **p** | **𝛽** | **[95% CI]** | **p** |
| Intercept | 9.28 | [7.38, 11.18] | **<0.001** | 7.91 | [7.81, 8.02] | **<0.001** |
| Age | -0.08 | [-0.12, -0.04] | **<0.001** | -0.02 | [-0.02, -0.02] | **<0.001** |
| Sex | 0.13 | [-0.52, 0.77] | 0.701 | -0.04 | [-0.08, 0.00] | **0.042** |
| BMI | -0.21 | [-0.78, 0.35] | 0.459 | 0.01 | [-0.02, 0.05] | 0.483 |
| Race: Black | 1.08 | [0.22, 1.94] | **0.015** | 0.06 | [0.01, 0.12] | **0.018** |
| Race: Other | 0.44 | [-0.41, 1.30] | 0.308 | -0.02 | [-0.07, 0.04] | 0.531 |
| CRP (µg/mL) | 0.18 | [-0.04, 0.41] | 0.109 | 0.00 | [-0.01, 0.01] | 0.690 |
| ICAM1 (ng/mL) | 0.00 | [0.00, 0.01] | 0.598 | 0.00 | [0.00, 0.00] | 0.989 |
| IL-6 (pg/mL) | 0.12 | [-0.04, 0.27] | 0.140 | 0.00 | [-0.01, 0.01] | 0.978 |
| IL-8 (pg/mL) | -0.06 | [-0.24, 0.12] | 0.507 | 0.00 | [0.00, 0.01] | 0.509 |
| Leptin (pg/mL) | 1.70E-05 | [0.00, 0.00] | **0.045** | 0.00E+00 | [0.00, 0.00] | 0.260 |
| MCP1 (pg/mL) | 0.00 | [0.00, 0.01] | 0.341 | 0.00 | [0.00, 0.00] | 0.446 |
| TNFα (pg/mL) | 0.00 | [-0.14, 0.14] | 0.981 | 0.00 | [-0.01, 0.01] | 0.992 |

**Table S12: General linear models testing associations between TL and individual F2-isoprostanes.** Coefficients reflect kb difference in TL for each one-year increase in chronological age or unit increase in F2-isoprostane levels (all in units of ng/mg creatinine). Coefficients for BMI, Sex, and Race are kb difference relative to reference groups of Lean BMI, males, and Whites. Significant effects indicated in bold.

|  | **aTL** | | | **DNAmTL** | | |
| --- | --- | --- | --- | --- | --- | --- |
|  | **𝛽** | **[95% CI]** | **p** | **𝛽** | **[95% CI]** | **p** |
| Intercept | 9.77 | [8.06, 11.47] | **<0.001** | 7.89 | [7.79, 7.98] | **<0.001** |
| Age | -0.08 | [-0.12, -0.05] | **<0.001** | -0.02 | [-0.02, -0.02] | **<0.001** |
| Sex | 0.32 | [-0.32, 0.95] | 0.330 | -0.04 | [-0.08, 0.00] | **0.042** |
| BMI | -0.12 | [-0.69, 0.44] | 0.674 | 0.01 | [-0.02, 0.05] | 0.520 |
| Race: Black | 1.18 | [0.33, 2.04] | **0.007** | 0.06 | [0.01, 0.12] | **0.016** |
| Race: Other | 0.47 | [-0.38, 1.32] | 0.279 | -0.01 | [-0.07, 0.04] | 0.577 |
| 2,3-dinor-iPF2α- III | 0.22 | [-0.12, 0.55] | 0.202 | -0.01 | [-0.02, 0.01] | 0.256 |
| iPF2α-III | -1.62 | [-3.07, -0.17] | **0.030** | 0.04 | [-0.02, 0.10] | 0.152 |
| iPF2α-VI | -0.02 | [-0.18, 0.13] | 0.748 | 0.00 | [-0.01, 0.00] | 0.667 |
| 8,12-iso-iPF2α-VI | 0.06 | [-0.05, 0.17] | 0.299 | 0.00 | [0.00, 0.01] | 0.446 |

**Table S13: Change scores for TL measurements for the full sample and each intervention group at baseline and follow up. Top Panel**: Summary statistics of aTL measurements generated using qPCR. **Bottom Panel**: Summary statistics of DNAmTL measurements generated using DNA methylation. Values shown are Mean (Standard Deviation). Maintenance Phase refers to TL attrition between 12- and 24- month follow up assessments P-values report results of t-test for differences between AL and CR groups at each time point**.** Significant effects indicated in bold.

| ΔaTL | | | | |
| --- | --- | --- | --- | --- |
|  | **Full Sample** | **AL** | **CR** | **p** |
| 12 Month | 0.40 (1.36) | 0.13 (1.25) | 0.55 (1.40) | 0.056 |
| 24 Month | 0.44 (1.42) | 0.56 (1.40) | 0.37 (1.43) | 0.415 |
| Maintenance | 0.09 (1.28) | 0.38 (1.14) | -0.08 (1.32) | **0.027** |
| ΔDNAmTL | | | | |
|  | **Full Sample** | **AL** | **CR** | **P** |
| 12 Month | 0.01 (0.05) | 0.003 (0.06) | 0.02 (0.05) | 0.068 |
| 24 Month | 0.04 (0.05) | 0.03 (0.05) | 0.04 (0.05) | 0.210 |
| Maintenance | 0.02 (0.05) | 0.03 (0.06) | 0.02 (0.05) | 0.623 |

**Table S14: Sensitivity models for treatment effect on the treated (TOT) analyses testing the impact of caloric restriction on change in TL**. Coefficients reflect kb difference in TL attrition associated with a 20% increase in CR, independent of group status. Maintenance Phase coefficients refer to associations with TL attrition between 12- and 24- month follow up assessments. Positive values reflect faster TL attrition between assessments, while negative values reflect slower TL attrition between assessments. Significant effects indicated in bold.

| Panel A: TOT Adjusted for Oxidative Stress and Inflammation | | | | | |  |
| --- | --- | --- | --- | --- | --- | --- |
|  | **aTL** | | | **DNAmTL** | | |
|  | **𝛽** | **[95% CI]** | **p** | **𝛽** | **[95% CI]** | **p** |
| Baseline to 12 Months | 0.565 | [0.024, 1.105] | **0.042** | 0.028 | [0.005, 0.051] | **0.020** |
| Baseline to 24 Months | -0.062 | [-0.731, 0.608] | 0.857 | 0.020 | [-0.007, 0.047] | 0.150 |
| Maintenance Phase | -0.843 | [-1.659, -0.026] | **0.045** | -0.012 | [-0.046, 0.023] | 0.510 |
| Panel B: TOT Adjusted for CBC Immune Cell Proportions | | | | | | |
|  | **aTL** | | | **DNAmTL** | | |
|  | **𝛽** | **[95% CI]** | **p** | **𝛽** | **[95% CI]** | **p** |
| Baseline to 12 Months | 0.553 | [-0.040, 1.145] | 0.070 | 0.021 | [-0.005, 0.046] | 0.119 |
| Baseline to 24 Months | -0.136 | [-0.878, 0.606] | 0.720 | 0.020 | [-0.009, 0.048] | 0.184 |
| Maintenance Phase | -0.879 | [-1.855, 0.096] | 0.080 | -0.003 | [-0.043, 0.038] | 0.901 |
| Panel C: TOT Adjusted for DNAm Immune Cell Proportions | | | | | | |
|  | **aTL** | | | **DNAmTL** | | |
|  | **𝛽** | **[95% CI]** | **p** | **𝛽** | **[95% CI]** | **p** |
| Baseline to 12 Months | 0.548 | [-0.019, 1.115] | 0.060 | 0.029 | [0.005, 0.053] | **0.020** |
| Baseline to 24 Months | -0.198 | [-0.872, 0.475] | 0.565 | 0.025 | [-0.002, 0.052] | 0.068 |
| Maintenance Phase | -0.727 | [-1.531, 0.077] | 0.079 | -0.013 | [-0.047, 0.021] | 0.457 |

**Table S15: TRN Reporting Guidelines**

| **ITEM** | **DESCRIPTION** | |
| --- | --- | --- |
| **Sample Type, Storage, Extraction, and Integrity** | | |
| Sample type | EDTA packed cells collected at three locations (Washington University, Pennington Biomedical Research Center, Tufts University) were shipped to University of Vermont for from a period of 2008 to 2011. | |
| DNA extraction method | DNA was extracted in batches as samples were receive using Puregene kits (Qiagen). | |
| DNA storage conditions, including freeze-thaw cycles | DNA was stored at 4°C after extraction at the CALERIE™ Biorepository at the University of Vermont. In 2020 DNA was shipped on dry ice to the Shalev Lab at Pennsylvania State University (PSU) for telomere length analysis. On average there were 9.74 years between exaction and shipment. On average there were four freeze-thaws for DNA samples between extraction and the qPCR assay. The first thaw was conducted to confirm receipt and reorganize samples upon arrival to PSU. The second thaw was done to determine dsDNA concentration using the Agilent 2200 TapeStation. The third thaw was needed to perform a dilution for the qPCR assay. The final thaw occurred when the sample was assayed for telomere length. Samples needing to be reassessed on qPCR assays (n=59; 10.29%) were thawed one additional time. DNA samples were stored for an average of 2.41 months between the TapeStation assay and qPCR assay. | |
| Method of documenting DNA quality and integrity | dsDNA concentration and quality were quantified for all samples using the Agilent 4150 TapeStation with mean dsDNA=309.59 ng/uL. DNA quality was assessed using the DNA Integrity Number with mean_DIN_=9.40, indicating intact, high quality DNA. No exclusionary criteria was imposed prior to assays. | |
| Percentage of samples specifically tested for DNA quality and integrity | All samples were subjected to quality control via evaluation of the DNA Integrity Number. | |
| **qPCR Assay** | | |
| Method (qPCR, MMqPCR, aTL, etc.) | qPCR assays to calculate absolute telomere length (aTL) were structured such that each assay comprised two qPCR runs, one run quantifying telomere content in kilobases (T) and a second run quantifying genome copy number (S) using the single copy gene *IFNB1.* The two runs (T & S) were always performed on the same day using the same DNA aliquot which was stored at 4°C between runs (~2.5 hours). Each run hosted triplicate reactions of 22 samples, 6 standards, 5 positive controls, and 1 no template control on 100 well disks.  A total of 32 qPCR assays were performed across a period of 24 days from 7/11/2022 to 8/04/2022 for analysis of all samples. | |
| PCR machine type | Qiagen Rotor-Gene Q using 100 well disks | |
| Source of master mix and reagents, and final reaction volume | The final reaction mix for the telomeric and *IFNB1* reactions contains 1x QuantiTect SYBR Green Master Mix (Qiagen), 0.2U Uracil Glycosylase (Thermo Fisher Scientific), 0.1 uM forward-reverse primer pair, and 6 ng DNA in a 20 uL reaction.  Primers are purchased from IDT in as pre-mixed pairs (RxnReady Primer Pool) with HPLC purification and 10uM concentration in IDTE Buffer pH 8.0. | |
| Telomere primer sequences and concentration | Forward Primer: 5'-CGG TTT GTT TGG GTT TGG GTT TGG GTT TGG GTT TGG GTT-3′  Reverse Primer: 5'-GGC TTG CCT TAC CCT TAC CCT TAC CCT TAC CCT TAC CCT-3′ | |
| Single copy gene name, primer sequences, and concentration | *IFNB1* Forward Primer: 5’-TGG CAC AAC AGG TAG TAG GCG ACA C-3’  *IFNB1* Reverse Primer: 5’-GCA CAA CAG GAG AGC AAT TTG GAG GA-3’ | |
| Full PCR program description including temperature, times, and cycle numbers | 50°C – 2min  95°C – 15min |  |
|  | 95°C – 15s | 40 cycles |
|  | 55°C for 1 min with data acquisition |  |
|  | Melt 60°C to 99°C rising 1°C per step with 5 sec per step | |
| PCR efficiency of single copy gene and telomere primers | Telo: R^2^ = 0.9975; Efficiency=2.03  IFNB1: R^2^ = 0.9985; Efficiency=2.04 | |
| Source and concentration of control samples and standard curve | 4 positive controls were randomly selected from within the sample to control for variation across T and S runs. The final control sample was comprised of DNA extracted from the Jurkat cell line (ThermoFisher), which is known to have short telomere length (<6kb). Standards consisted of double stranded oligomers purchased from IDT as lyophilized pellet with PAGE purification.  Standard curves for T runs consisted of 84 bp double stranded oligomer comprised of 16 copies of canonical telomere repeat. Telomere Standard A had concentration 0.10 ng/uL, which equates to 5.86e+08 kb telomeric DNA when 6uL is used in the qPCR assay. A series of 1/10 serial dilutions were performed to generate a total of 6 standards for each T run comprising a range of 5.86e+08 to 5.86e+03 kb telomeric DNA.  Standard curves for S runs consisted of 83 bp double stranded oligomer corresponding to the region of IFNB1 genomic DNA flanked by IFNB1 primers. IFNB1 Standard 1 had concentration 0.00033 ng/uL, which equates to 1.18e+07 diploid genomes when 6uL is used in the qPCR assay. A series of 1/10 serial dilutions were performed to generate a total of 6 standards for each S run comprising a range of 1.18e+07 to 1.18e+02 diploid genome copies. | |
| Telomere Standard Oligomer Sequences | Sense: 5’-CCC TAA CCC TAA CCC TAA CCC TAA CCC TAA CCC TAA CCC TAA CCC TAA CCC TAA CCC TAA CCC TAA CCC TAA CCC TAA CCC TAA-3’  Anti-sense: 5’-TTA GGG TTA GGG TTA GGG TTA GGG TTA GGG TTA GGG TTA GGG TTA GGG TTA GGG TTA GGG TTA GGG TTA GGG TTA GGG TTA GGG-3’ | |
| *IFNB1* Standard Oligomer Sequences | Sense: 5-GCA CAA CAG GAG AGC AAT TTG GAG GAG ACA CTT GTT GGT CAT GTT GAC AAC ACG AAC AGT GTC GCC TAC TAC CTG TTG TGC CA-3’  5’-TGG CAC AAC AGG TAG TAG GCG ACA CTG TTC GTG TTG TCA ACA TGA CCA ACA AGT GTC TCC TCC AAA TTG CTC TCC TGT TGT GC-3’ | |
| **Data Analysis** | | |
| Mean and standard deviation or median range of telomere lengths | See **Table S5.** | |
| Number of sample replicates | Each sample was assessed for T and S on a single run with three replicates within the run. If the sample did not pass quality control criteria described below it was run a second time. | |
| Level of independence of replicates | Replicates were drawn from the same DNA aliquot (i.e., the same tube). | |
| Analytic method, considering replicate measurements, to determine final length | Estimates of kb telomeric DNA and genome copy number were calculated automatically based on the alignment of each sample with the standard curve. When applicable, estimates for the no template control were subtracted from estimates of the analytical samples prior to calculating aTL values. The average kb telomeric DNA estimates and genome copy number estimates across triplicate measurements were used to calculate aTL values.  $aTL=\frac{Estimated kb Telomeric DNA}{Estimated Genome Copy Number\times92}$ | |
| Method of accounting for variation between replicates | When the coefficient of variation across triplicate estimates of telomere content or genome copy number was greater than 15%, replicate estimates were evaluated based upon their deviation from mean across triplicates. If one replicate deviated from the mean by more than 15% it was considered an outlier and the mean was recalculated using two replicates. Excepting samples that were rerun or failed, an average of 5.25 T replicates and 1.16 S replicates were dropped per run (*in this case aTL values were calculated using the average across duplicate measures*).  In the case where coefficient of variation across replicates was still greater than 15% after removal of a single outlier, or was greater than 15% without a clear outlier defined by the criteria above, the sample was reassessed for both telomere content and genome copy number, and subjected to the same quality control evaluation. A total of 59 samples (10.99%) were rerun a second time. Of these 59 samples, two were rerun a third time, and a third sample was retained despite exhibiting a high CV (30.12%).  In the case where a sample was amplified abnormally on both attempts it was removed from the sample and not assayed a third time. This occurred for 1 (0.17%) sample. | |
| Method of accounting for well position effects within plates | The unique rotary design of the Rotor Gene Q is optimized to minimize well position effects. As such no accounting for well position effects was performed. | |
| Method of accounting for between plate effects | To control for inter-assay variability, the telomeric content and genome copy number were assessed for five control samples on each T run and each S run. For each run, the estimated telomeric content and genome copy number were divided by the average estimated telomeric content and genome copy number for all runs to get a normalizing factor for that sample on a given run. This was done for all controls to get an average normalizing factor for that run. Estimates for analytical samples were then divided by the normalization factor for a given run. In this manner the average intra-run CV across replicate kb telomeric DNA estimates and genome copy number estimates was 5.82% and 3.86% respectively. The average inter-assay CV for aTL estimates of 5 control samples across all 32 assays was 7.54%. | |
| % of samples repeated and % of samples failing QC and excluding from further analyses | 59/573 = 10.29% of samples repeated  1/573 = 0.17% of samples failed QC and excluded from analyses. | |
| Acceptable range of PCR efficiency for single copy gene and telomere primers | 1.90 – 2.10 (5*% variation*) | |
| ICCs of samples/study groups to address variability | A selection of 88 samples with duplicate aTL values were utilized for the purposes of calculating the ICC. These samples were balanced such that they included at least two samples from each of the first 26 assays conducted to analyze each sample. ICCs were calculated at the level of aTL values using the *rpt* function in R using a Gaussian data distribution. The global ICC was 0.834 and was similar between those in the ad libitum (0.859) and caloric restriction groups (0.821). The ICC remained unchanged after covariate control for sampling occasion (i.e., baseline, 12 months, 24 months; ICC= 0.834).  Due to the use of biobanked DNA, we were unable to determine ICC across repeatedly extracted samples as recommended by the Telomere Research Network. For this estimate, we refer readers to other work from our lab using aTL measurements derived using the same protocol (Wolf et al., 2024). In this work, the ICC across repeated extractions was 0.826. | |
| T/S ratio transformed to a z-score prior before comparison across methods/studies | N/A. No comparison across studies was conducted. | |
| How samples nested within families were accounted for | Samples from the same individual were run on the same plate except in cases when a single sample from a given individual needed to be rerun due to high intra-assay CV. | |

**Table S16: Factor loading scores for F2-isoprostanes**

Factor loading scores for PC1 were extracted and utilized in models to represent an individual aggregate oxidative stress status

| **Biomarker** | **PC1** | **PC2** | **PC3** | **PC4** |
| --- | --- | --- | --- | --- |
| 2,3-dinor-iPF2α III | 0.49 | 0.52 | -0.57 | 0.40 |
| IPF2α III | 0.49 | 0.37 | 0.79 | 0.03 |
| iPF2α VI | 0.54 | -0.15 | -0.23 | -0.80 |
| 8,12-iso-iPF2α VI | 0.49 | 0.52 | -0.57 | 0.40 |
| *Eigenvalue* | 1.71 | 0.72 | 0.60 | 0.44 |
| *Proportion of Variance* | 73% | 13% | 9% | 5% |

**Table S17: Distribution of inflammatory biomarkers utilized in principal component analysis**

Prior to performing principal component analysis (PCA) inflammatory biomarkers distributions were screened for skew and kurtosis. Panel A shows features of the original biomarker distribution. Panel B shows biomarker distributions following removal of outliers. Panel C shows biomarker distribution following imputation of missing values. A total of 282 (7.17%) of values were imputed across the 7 biomarkers. Imputation was performed for samples with available data for at least 4 of the 7 biomarkers using the *mice* package in R. SD= standard deviation; SE= standard error.

| Panel A: Raw Distribution | | | | | | |
| --- | --- | --- | --- | --- | --- | --- |
| **Biomarker** | **N** | **Mean** | **SD** | **Skew** | **Kurtosis** | **SE** |
| CRP (µg/mL) | 564 | 1.30 | 2.84 | 7.28 | 73.42 | 0.12 |
| ICAM1 (ng/mL) | 561 | 157.38 | 40.71 | -0.68 | 2.17 | 1.72 |
| IL-6 (pg/mL) | 554 | 1.91 | 1.86 | 2.83 | 9.26 | 0.08 |
| IL-8 (pg/mL) | 564 | 1.81 | 1.89 | 8.31 | 109.77 | 0.08 |
| Leptin (pg/mL) | 564 | 12,866.39 | 12,281.71 | 1.83 | 4.09 | 517.15 |
| MCP1 (pg/mL) | 564 | 112.14 | 61.15 | 5.43 | 60.96 | 2.57 |
| TNFα (pg/mL) | 562 | 3.08 | 1.51 | 4.22 | 37.28 | 0.06 |
| Panel B: Outliers Removed | | | | | | |
| **Biomarker** | **N** | **Mean** | **SD** | **Skew** | **Kurtosis** | **SE** |
| CRP (µg/mL) | 499 | 0.62 | 0.57 | 1.44 | 1.70 | 0.03 |
| ICAM1 (ng/mL) | 514 | 162.68 | 27.52 | 0.10 | -0.13 | 1.21 |
| IL-6 (pg/mL) | 496 | 1.38 | 0.74 | 1.04 | 0.86 | 0.03 |
| IL-8 (pg/mL) | 526 | 1.47 | 0.74 | 0.74 | 0.07 | 0.03 |
| Leptin (pg/mL) | 534 | 10822.42 | 8660.36 | 1.02 | 0.31 | 374.77 |
| MCP1 (pg/mL) | 534 | 102.59 | 34.82 | 0.34 | -0.16 | 1.51 |
| TNFα (pg/mL) | 549 | 2.94 | 1.04 | 0.33 | -0.28 | 0.04 |
| Panel C: Outliers Imputed | | | | | | |
| **Biomarker** | **N** | **Mean** | **SD** | **Skew** | **Kurtosis** | **SE** |
| CRP (µg/mL) | 562 | 0.63 | 0.58 | 1.40 | 1.52 | 0.02 |
| ICAM1 (ng/mL) | 562 | 162.81 | 27.54 | 0.12 | -0.14 | 1.16 |
| IL-6 (pg/mL) | 562 | 1.40 | 0.75 | 1.00 | 0.68 | 0.03 |
| IL-8 (pg/mL) | 562 | 1.46 | 0.73 | 0.73 | 0.09 | 0.03 |
| Leptin (pg/mL) | 562 | 11035.27 | 8814.96 | 1.00 | 0.23 | 371.84 |
| MCP1 (pg/mL) | 562 | 102.78 | 35.2 | 0.34 | -0.16 | 1.48 |
| TNFα (pg/mL) | 562 | 2.95 | 1.05 | 0.31 | -0.30 | 0.04 |

**Table S18: Results of principal component analysis of inflammatory biomarkers**

Principal component analyses were conducted using data from all sample time points. Panel A shows factor loadings and eigenvalues for the original biomarker distribution. Panel B shows factor loadings and eigenvalues for the inflammatory biomarkers following removal of outliers. Panel C shows factor loadings and eigenvalues for the inflammatory biomarkers following imputation of missing biomarker values for samples with valid data for at least 4 of the 7 biomarkers. A total of 282 values were imputed (7.17%). The first principal component (PC1) was extracted for use in statistical analyses.

| Panel A: Raw Distribution | | | | | | | |
| --- | --- | --- | --- | --- | --- | --- | --- |
| **Biomarker** | **PC1** | **PC2** | **PC3** | **PC4** | **PC5** | **PC6** | **PC7** |
| CRP | 0.28 | -0.63 | 0.03 | -0.33 | 0.35 | 0.03 | 0.54 |
| ICAM1 | 0.33 | 0.28 | -0.17 | -0.71 | -0.06 | -0.48 | -0.20 |
| IL-6 | 0.32 | -0.35 | 0.55 | -0.07 | -0.63 | 0.11 | -0.25 |
| IL-8 | 0.33 | 0.19 | 0.57 | 0.35 | 0.47 | -0.42 | -0.05 |
| Leptin | 0.35 | -0.43 | -0.50 | 0.31 | 0.15 | -0.08 | -0.56 |
| MCP1 | 0.49 | 0.22 | -0.30 | 0.37 | -0.42 | -0.15 | 0.53 |
| TNF-α | 0.48 | 0.36 | 0.01 | -0.13 | 0.24 | 0.74 | -0.10 |
| *Eigenvalue* | 1.19 | 1.09 | 1.03 | 1.00 | 0.92 | 0.90 | 0.84 |
| *Proportion of Variance* | 20% | 17% | 15% | 14% | 12% | 12% | 10% |
| Panel B: Outliers Removed | | | | | | | |
| **Biomarker** | **PC1** | **PC2** | **PC3** | **PC4** | **PC5** | **PC6** | **PC7** |
| CRP | 0.41 | -0.40 | 0.26 | -0.37 | 0.09 | 0.62 | 0.26 |
| ICAM1 | 0.40 | -0.02 | -0.58 | -0.17 | 0.59 | -0.30 | 0.21 |
| IL-6 | 0.35 | -0.35 | -0.02 | 0.80 | 0.13 | 0.14 | -0.29 |
| IL-8 | 0.26 | 0.23 | 0.74 | 0.11 | 0.31 | -0.41 | 0.23 |
| Leptin | 0.24 | -0.58 | 0.06 | -0.26 | -0.42 | -0.56 | -0.20 |
| MCP1 | 0.46 | 0.34 | -0.19 | 0.21 | -0.60 | 0.01 | 0.49 |
| TNF-α | 0.46 | 0.47 | 0.03 | -0.26 | -0.04 | 0.12 | -0.69 |
| *Eigenvalue* | 1.26 | 1.16 | 1.03 | 0.94 | 0.90 | 0.84 | 0.78 |
| *Proportion of Variance* | 23% | 19% | 15% | 13% | 12% | 10% | 9% |
| Panel C: Outliers Imputed | | | | | | | |
| **Biomarker** | **PC1** | **PC2** | **PC3** | **PC4** | **PC5** | **PC6** | **PC7** |
| CRP | 0.42 | 0.34 | 0.28 | 0.47 | -0.17 | 0.47 | 0.40 |
| ICAM1 | 0.43 | 0.01 | -0.55 | 0.09 | -0.49 | -0.48 | 0.18 |
| IL-6 | 0.39 | 0.19 | -0.09 | -0.84 | 0.01 | 0.32 | 0.10 |
| IL-8 | 0.23 | -0.28 | 0.77 | -0.19 | -0.29 | -0.40 | 0.03 |
| Leptin | 0.35 | 0.58 | 0.11 | 0.10 | 0.26 | -0.29 | -0.61 |
| MCP1 | 0.42 | -0.36 | -0.06 | 0.10 | 0.74 | -0.18 | 0.31 |
| TNF-α | 0.37 | -0.55 | -0.10 | 0.14 | -0.17 | 0.41 | -0.57 |
| *Eigenvalue* | 1.29 | 1.12 | 1.01 | 0.94 | 0.90 | 0.84 | 0.80 |
| *Proportion of Variance* | 24% | 18% | 14% | 13% | 11% | 10% | 9% |

**References**

Aryee, M. J., Jaffe, A. E., Corrada-Bravo, H., Ladd-Acosta, C., Feinberg, A. P., Hansen, K. D., & Irizarry, R. A. (2014). Minfi: a flexible and comprehensive Bioconductor package for the analysis of Infinium DNA methylation microarrays. *Bioinformatics*, *30*(10), 1363-1369. <https://doi.org/10.1093/bioinformatics/btu049>

Bang, H., & Davis, C. E. (2007). On estimating treatment effects under non-compliance in randomized clinical trials: are intent-to-treat or instrumental variables analyses perfect solutions? *Stat Med*, *26*(5), 954-964. <https://doi.org/10.1002/sim.2663>

Davis, S. (2021). *methylumi: Handle Illumina methylation data*. In (Version 3.13)

Hastings, W. J., Eisenberg, D. T. A., & Shalev, I. (2020). Uninterruptible Power Supply Improves Precision and External Validity of Telomere Length Measurement via qPCR. *Exp Results*, *1*. <https://doi.org/10.1017/exp.2020.58>

Hastings, W. J., Etzel, L., Heim, C. M., Noll, J. G., Rose, E. J., Schreier, H. M. C., Shenk, C. E., Tang, X., & Shalev, I. (2022). Comparing qPCR and DNA methylation-based measurements of telomere length in a high-risk pediatric cohort. *Aging (Albany NY)*, *14*(2), 660-677. <https://doi.org/10.18632/aging.203849>

Higgins-Chen, A. T., Thrush, K. L., Wang, Y., Minteer, C. J., Kuo, P. L., Wang, M., Niimi, P., Sturm, G., Lin, J., Moore, A. Z., Bandinelli, S., Vinkers, C. H., Vermetten, E., Rutten, B. P. F., Geuze, E., Okhuijsen-Pfeifer, C., van der Horst, M. Z., Schreiter, S., Gutwinski, S., . . . Levine, M. E. (2022). A computational solution for bolstering reliability of epigenetic clocks: Implications for clinical trials and longitudinal tracking. *Nat Aging*, *2*(7), 644-661. <https://doi.org/10.1038/s43587-022-00248-2>

Huber, W., Carey, V. J., Gentleman, R., Anders, S., Carlson, M., Carvalho, B. S., Bravo, H. C., Davis, S., Gatto, L., Girke, T., Gottardo, R., Hahne, F., Hansen, K. D., Irizarry, R. A., Lawrence, M., Love, M. I., MacDonald, J., Obenchain, V., Oles, A. K., . . . Morgan, M. (2015). Orchestrating high-throughput genomic analysis with Bioconductor. *Nat Methods*, *12*(2), 115-121. <https://doi.org/10.1038/nmeth.3252>

Lehne, B., Drong, A. W., Loh, M., Zhang, W., Scott, W. R., Tan, S. T., Afzal, U., Scott, J., Jarvelin, M. R., Elliott, P., McCarthy, M. I., Kooner, J. S., & Chambers, J. C. (2015). A coherent approach for analysis of the Illumina HumanMethylation450 BeadChip improves data quality and performance in epigenome-wide association studies. *Genome Biol*, *16*(1), 37. <https://doi.org/10.1186/s13059-015-0600-x>

Martin, C. K., Hochsmann, C., Dorling, J. L., Bhapkar, M., Pieper, C. F., Racette, S. B., Das, S. K., Redman, L. M., Kraus, W. E., Ravussin, E., & Group, C. P. S. (2022). Challenges in defining successful adherence to calorie restriction goals in humans: Results from CALERIE 2. *Exp Gerontol*, *162*, 111757. <https://doi.org/10.1016/j.exger.2022.111757>

O'Callaghan, N. J., & Fenech, M. (2011). A quantitative PCR method for measuring absolute telomere length. *Biol Proced Online*, *13*, 3. <https://doi.org/10.1186/1480-9222-13-3>

Ramaker, M. E., Corcoran, D. L., Apsley, A. T., Kobor, M. S., Kraus, V. B., Kraus, W. E., Lin, D. T. S., Orenduff, M. C., Pieper, C. F., Waziry, R., Huffman, K. M., & Belsky, D. W. (2022). Epigenome-wide Association Study Analysis of Calorie Restriction in Humans, CALERIETM Trial Analysis. *J Gerontol A Biol Sci Med Sci*, *77*(12), 2395-2401. <https://doi.org/10.1093/gerona/glac168>

Salas, L. A., Koestler, D. C., Butler, R. A., Hansen, H. M., Wiencke, J. K., Kelsey, K. T., & Christensen, B. C. (2018). An optimized library for reference-based deconvolution of whole-blood biospecimens assayed using the Illumina HumanMethylationEPIC BeadArray. *Genome Biol*, *19*(1), 64. <https://doi.org/10.1186/s13059-018-1448-7>

Sussman, J. B., & Hayward, R. A. (2010). An IV for the RCT: using instrumental variables to adjust for treatment contamination in randomised controlled trials. *BMJ*, *340*, c2073. <https://doi.org/10.1136/bmj.c2073>

Wolf, S. E., Hastings, W. J., Ye, Q., Etzel, L., Apsley, A. T., Chiaro, C., Heim, C. C., Heller, T., Noll, J. G., & Schreier, H. M. (2024). Cross-tissue comparison of telomere length and quality metrics of DNA among individuals aged 8 to 70 years. *PLoS One*, *19*(2), e0290918.
